# Condensin II and GAIT complexes cooperate to restrict LINE-1 retrotransposition in epithelial cells

**DOI:** 10.1371/journal.pgen.1007051

**Published:** 2017-10-13

**Authors:** Jacqueline R. Ward, Kommireddy Vasu, Emily Deutschman, Dalia Halawani, Peter A. Larson, Dongmei Zhang, Belinda Willard, Paul L. Fox, John V. Moran, Michelle S. Longworth

**Affiliations:** 1 Department of Cellular and Molecular Medicine, Lerner Research Institute, Cleveland Clinic, Cleveland, OH, United States of America; 2 Department of Human Genetics, University of Michigan School of Medicine, Ann Arbor, MI, United States of America; 3 Proteomics Core, Lerner Research Institute, Cleveland Clinic, Cleveland, OH, United States of America; 4 Department of Internal Medicine, University of Michigan School of Medicine, Ann Arbor, MI, United States of America; MIT, UNITED STATES

## Abstract

LINE-1 (L1) retrotransposons can mobilize (retrotranspose) within the human genome, and mutagenic *de novo* L1 insertions can lead to human diseases, including cancers. As a result, cells are actively engaged in preventing L1 retrotransposition. This work reveals that the human Condensin II complex restricts L1 retrotransposition in both non-transformed and transformed cell lines through inhibition of L1 transcription and translation. Condensin II subunits, CAP-D3 and CAP-H2, interact with members of the Gamma-Interferon Activated Inhibitor of Translation (GAIT) complex including the glutamyl-prolyl-tRNA synthetase (EPRS), the ribosomal protein L13a, Glyceraldehyde 3-phosphate dehydrogenase (GAPDH), and NS1 associated protein 1 (NSAP1). GAIT has been shown to inhibit translation of mRNAs encoding inflammatory proteins in myeloid cells by preventing the binding of the translation initiation complex, in response to Interferon gamma (IFN-γ). Excitingly, our data show that Condensin II promotes complexation of GAIT subunits. Furthermore, RNA-Immunoprecipitation experiments in epithelial cells demonstrate that Condensin II and GAIT subunits associate with L1 RNA in a co-dependent manner, independent of IFN-γ. These findings suggest that cooperation between the Condensin II and GAIT complexes may facilitate a novel mechanism of L1 repression, thus contributing to the maintenance of genome stability in somatic cells.

## Author summary

LINE-1 (L1) retrotransposons mobilize within the human genome and L1 insertions are implicated in the development of various cancers. This work identifies the Condensin II complex as a novel repressor of L1 expression and retrotransposition in human primary and transformed epithelial cells. Proteomics analyses reveal interactions between Condensin II subunits, CAP-D3 and CAP-H2, and Gamma-Interferon Activated Inhibitor of Translation (GAIT) complex subunits. GAIT inhibits translation of inflammatory mRNAs in monocytes by binding to 3’ UTRs and preventing translation initiation in response to IFN-γ. We demonstrate that CAP-D3 promotes GAIT complex formation. CAP-D3 and GAIT members associate with L1 RNA in a co-dependent manner, to facilitate repression of L1 translation and retrotransposition, independent of IFN-γ. This work describes the unique cooperation between two multi-subunit complexes, canonically involved in very different essential cellular functions, to inhibit L1 expression through a novel mechanism, thus maintaining its repression in somatic cells.

## Introduction

Retrotransposons are DNA elements that can mobilize (retrotranspose) throughout the genome via an RNA intermediate [1]. Long INterspersed Element-1 (LINE-1s or L1s), a type of non-long terminal repeat (LTR) retrotransposon, comprises ~17% of the human genome and is the only currently active, autonomous human retrotransposon [2, 3]. The mobility of L1 is dependent on transcription and translation of its encoded proteins and subsequent insertion into a novel region in the genome [reviewed in [3, 4]]. Through their mobilization and insertion, L1s have a profound capacity for interfering with genome stability by creating DNA deletions, insertions and rearrangements [3, 5–8]. L1 retrotransposons can also alter gene expression by disrupting exons, by inducing mis-splicing or premature poly-adenylation, and by altering the transcriptional profile of a gene due to the anti-sense promoter within the L1 5’UTR [9–12]. *De novo* insertions of L1 copies have been implicated in diseases including hemophilia A, Duchenne muscular dystrophy, and various cancers [13–21].

L1 contains two open-reading frames (ORF1 and ORF2), which, upon translation, give rise to two distinct retrotransposition-essential proteins, ORF1p and ORF2p [22–24]. ORF1 encodes a 40 kDa RNA-binding protein that has nucleic acid chaperone capabilities [25–29]. ORF2 encodes a 150 kDa protein with demonstrated endonuclease and reverse transcriptase activities [23, 30–34]. ORF1p and ORF2p preferentially bind to their encoding mRNA and form ribonucleoprotein (RNP) complexes [25, 35–39]. The L1 RNP gains access into the nucleus either by active import or passively, perhaps during mitotic nuclear envelope breakdown [40, 41]. The ORF2p endonuclease cleaves genomic DNA to expose a 3'-hydroxyl residue that is used as a primer by the L1 reverse transcriptase to copy the L1 RNA, a mechanism that has been termed target-primed reverse transcription (TPRT), leading to a new L1 insertion at a different site in the genome [23, 42, 43].

Due to the deleterious consequences associated with L1 retrotransposition, human cells have evolved various mechanisms to restrict retrotransposition. These include, but are not limited to, L1 DNA methylation, mutation and/or degradation of the L1 RNA, and inhibition of L1 RNP formation [44–53]. Interestingly, multiple reports also demonstrate roles for cellular proteins in targeting L1 RNAs to prevent accumulation of L1-essential proteins [54–59]. For example, downregulation of L1 RNA by the ribonuclease, RNase L, results in reduced expression of ORF1p and ORF2p [60]. Additionally, SAM domain and HD domain 1 (SAMHD1), a DNTPase which also possesses ribonuclease activity, enhances assembly of cytoplasmic stress granules that sequester L1 RNPs and prevent their retrotransposition [35, 55, 61].

Recently, our lab identified the DNA organizing complex Condensin II as being necessary for the inhibition of retrotransposition in *Drosophila* cells and tissues [62]. Condensins are multi-subunit protein complexes that play fundamental roles in chromosome organization and segregation during mitosis [63–69]. Eukaryotic cells possess two Condensins, known as Condensin I and Condensin II [69, 70]. These complexes differ not only in their components, but also in the fact that Condensin II can be found in the nucleus throughout the cell cycle, whereas Condensin I comes into contact with DNA after nuclear envelope breakdown in mitosis [65].

Condensin II contains two Structural Maintenance of Chromosomes (SMC) subunits, SMC2 and SMC4, which form the enzymatic (ATPase) and structural core of the complex to constrain positive supercoils. Condensin II also contains three non-SMC subunits or Chromatin Associated Proteins (CAP), CAP-D3, CAP-G2, and the kleisin family protein, CAP-H2. CAP-D3 and CAP-G2 contain HEAT repeats, a repeat motif implicated in protein-protein interactions [71]. Together these CAP proteins stabilize the holocomplex and promote the ATPase activity when they are phosphorylated [72, 73].

In addition to its canonical function during mitosis, the Condensin II complex also plays a role in DNA organization during interphase [74–78]. Our recent studies in *Drosophila* showed that dCAP-D3 and dCAP-H2 regulate positioning of retrotransposon-containing loci within the nucleus [62]. Our data also demonstrated a role for Condensin II in maintaining repression of retrotransposon mRNA levels to inhibit retrotransposition, but the mechanism of how this occurs is still unknown. Since Condensin II subunits are conserved between flies and human cells, the question arises as to whether Condensin II represses L1 retrotransposition in human cells. Interestingly, mutations in Condensin II proteins have been identified in different types of somatic cancers [79], but whether these mutations or the resulting loss of protein expression directly lead to genomic instability, and the mechanism involved, are not well understood.

Here, we report that the Condensin II complex represses L1 retrotransposition in both non-transformed and transformed human epithelial cell lines. Excitingly, our data suggest that Condensin II subunits are necessary to repress L1s transcriptionally and post-transcriptionally. We have identified interactions between Condensin II subunits and members of the GAIT complex, a complex identified to repress translation in monocytes. GAIT has been shown to inhibit translation of mRNAs encoding inflammatory proteins in myeloid cells by binding the mRNAs and preventing binding of translation initiation complexes in response to IFN-γ [80–82]. While the GAIT subunit, EPRS, was previously shown to associate with ZAP (ZC3HAV1) as a component of the L1 RNP [59, 83], a clear role for EPRS and/or GAIT in the regulation of L1 activity was never determined. Our data demonstrate that the Condensin II subunits interact with GAIT subunits and L1 RNAs in a co-dependent manner. Loss of GAIT subunit expresson results in increased L1 expression and retrotransposition. Finally, we provide evidence that Condensin II, in cooperation with GAIT, prevents binding of EIF4F and the 43S complex to L1 RNA and represses L1 translation in epithelial cells.

## Results

### Condensin II represses L1 retrotransposition in human cells

We previously observed that Condensin II represses retrotransposition in *Drosophila melanogaster* [62]. Given that CAP-D3/Condensin II depletion in human cells is associated with genome instability [84–86], and L1 retrotransposition can lead to genome instability in human cells [87, 88], we investigated whether a similar role for the complex in repressing L1 retrotransposition might also exist in human cells. We used an established cell culture assay to determine whether endogenous Condensin II subunits, CAP-D3 and CAP-H2, affect retrotransposition of the L1 retrotransposon. HT-29 colon epithelial adenocarcinoma cells expressing inducible Non-Target, CAP-D3, or CAP-H2 shRNAs [89] were transfected with a full-length, retrotransposition-competent L1 plasmid [22, 90, 91]. The L1 construct contains an indicator cassette in the 3′ end, which consists of an antisense copy of a neomycin phosphotransferase gene (*mneoI*) equipped with a heterologous promoter (Pr′) and polyadenylation signal. The *mneoI* cassette is disrupted by an intron that is in the same transcriptional orientation as the L1 retrotransposon. This orientation ensures that the reporter gene will only become activated and expressed if the retrotransposon RNA is reverse transcribed and integrated into genomic DNA [22, 88, 92]. The resultant G418-resistant foci serve as a readout of L1 retrotransposition efficiency.

**Fig 1:**
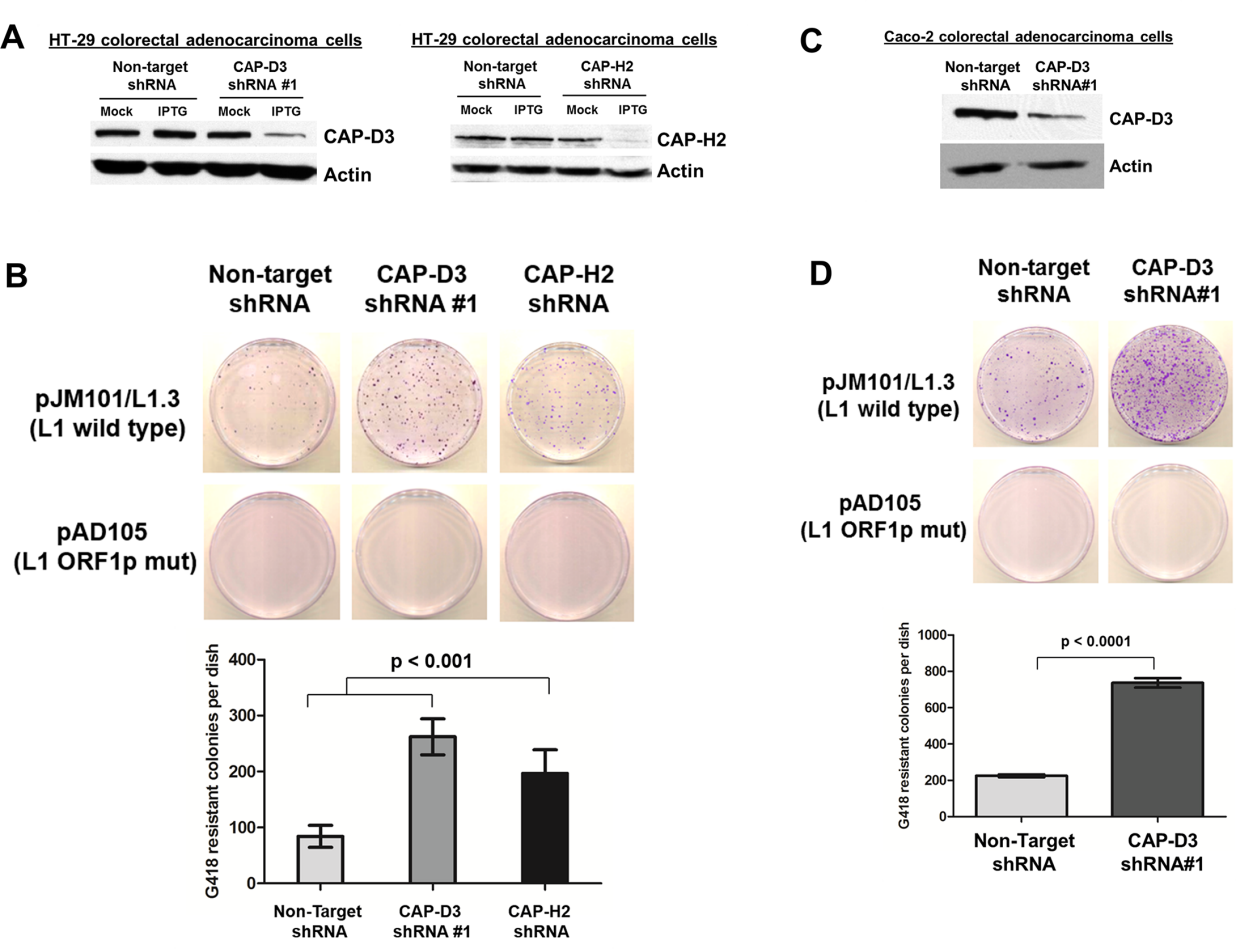
Condensin II represses LINE-1 retrotransposition in human cells. (A) Immunoblotting for CAP-D3 and CAP-H2 in HT-29 colorectal adenocarcinoma cells expressing inducible Non-Target (control), CAP-D3, or CAP-H2 shRNA. Cells were mock (PBS) or IPTG treated to induce shRNA expression. Actin was used as a loading control. (B) Retrotransposition assays involving full-length, retrotransposition competent (wild type) L1s (top row) or retrotransposition-defective ORF1p mutant L1s (bottom row) in HT-29 cells expressing Non-Target, CAP-D3 or CAP-H2 shRNAs. Crystal violet stained drug-resistant foci were quantified using ImagePro. (C) Immunoblotting for CAP-D3 in Caco2 colorectal adenocarcinoma cells expressing inducible Non-Target or CAP-D3 shRNA (similar to (A)). (D) Retrotransposition assays involving wild-type (top row) or mutant/defective L1s (bottom row) in Caco2 cells deficient for CAP-D3 (similar to (B)). P-values were calculated with a student t-test.

Retrotransposition assays revealed that decreased expression of CAP-D3 or CAP-H2 in IPTG-induced HT-29 cells resulted in significant increases in drug-resistant foci compared to Non-Target shRNA expressing cells (Fig 1A, Fig 1B, S1A Fig, S1B Fig). Mock (PBS) treated Non-Target and CAP-D3 shRNA expressing cells showed no significant difference in the amount of drug-resistant foci (S1C Fig). As a negative control, a retrotransposition-defective plasmid, which encodes a mutated version of ORF1p was transfected into HT-29 cells and this resulted in complete abrogation of drug-resistant foci, as expected (Fig 1B, S1B Fig) [35]. We observed a similar effect on retrotransposition in CAP-D3-deficient Caco-2 cells, another colon epithelial adenocarcinoma cell line (Fig 1C, Fig 1D). To monitor whether knockdown of CAP-D3 influences cell proliferation, we performed CyQUANT assays to measure the amount of DNA in Non-Target or CAP-D3 shRNA expressing cells over a 48 hour timecourse. We observed that reduced expression of CAP-D3 actually results in a significant decrease in cell proliferation by 48 hours; therefore, the increase in L1 retrotransposition observed is not due to an increase in cell proliferation (S2 Fig).

### Depletion of Condensin II results in increased levels of full length L1 RNA

**Fig 2:**
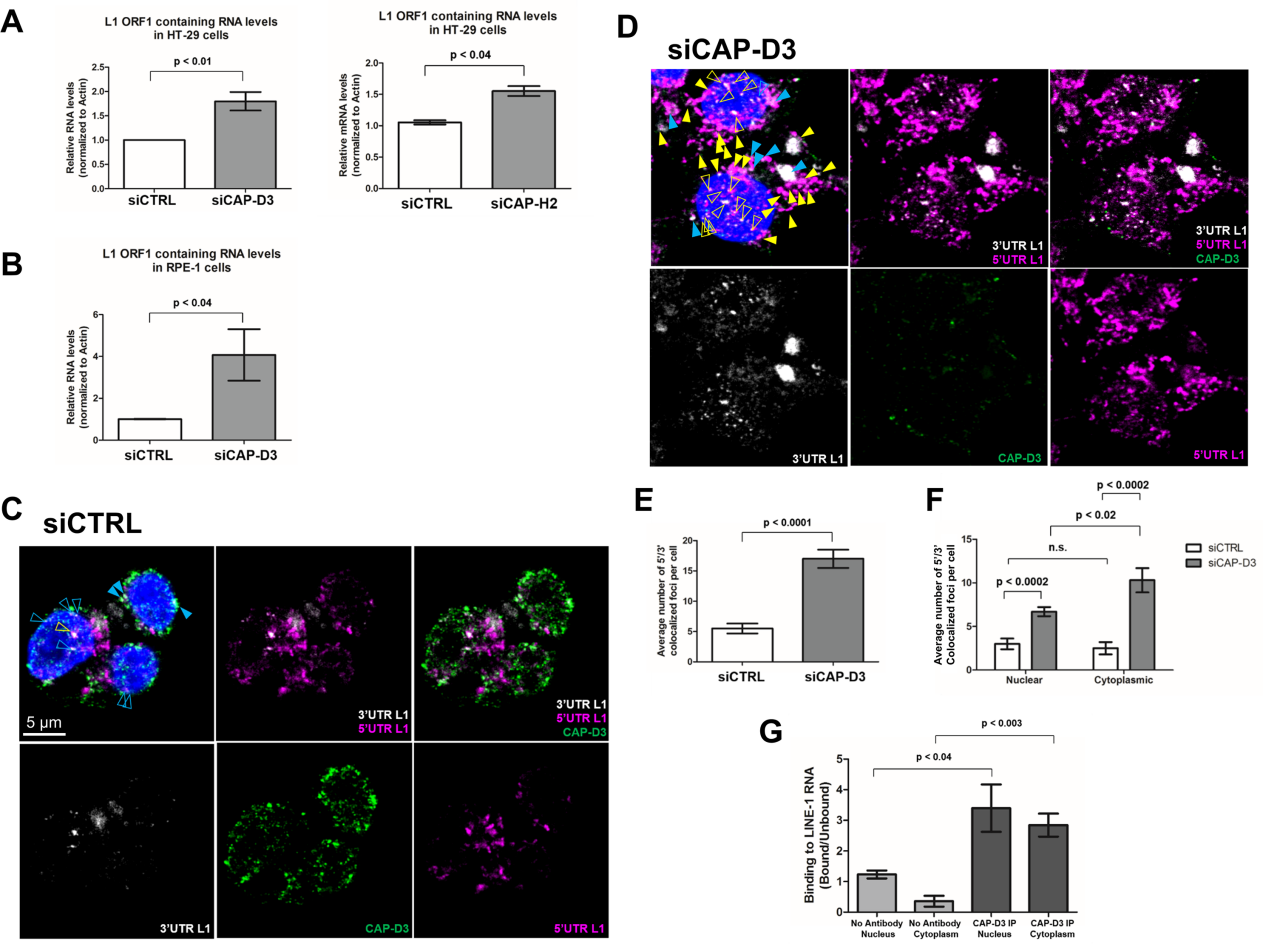
Depletion of the Condensin II subunit, CAP-D3, increases endogenous LINE-1 RNA. (A and B) qRT-PCR analysis of endogenous L1 RNA levels in HT-29 (A) or non-transformed RPE-1 (B) cells depleted of CAP-D3 or CAP-H2 by siRNA transfection compared to control siRNA transfected cells. Results were normalized to actin. (C and D) RNA FISH for L1 in HT-29 cells transfected with control siRNA (C) or CAP-D3 siRNA (D), combined with Immunostaining for CAP-D3 protein (green). Blue arrowheads indicate colocalization of foci containing both the 3’ UTR (white) and 5’ UTR (pink) of L1 RNA (full length L1 RNA) with CAP-D3. Yellow arrowheads indicate foci containing full length L1 RNA only. Open arrowheads signify nuclear localization of full length L1 RNA and filled arrowheads signify cytoplasmic localization. Nuclei were stained with DAPI (blue). (E) Quantitation of the average number of full length L1 RNA containing foci per cell (n = 10). (F) Quantitation of the average number of full length L1 RNA containing foci in the nucleus and cytoplasm (n = 10). (G) Quantitation of RNA-IP assays using CAP-D3 antibody or no antibody in fractionated HT-29 cell lysates. Binding of CAP-D3 was normalized to the signal intensity in the unbound fractions for each sample (n = 3). P-values were calculated with a student t-test.

The observed increase in L1 retrotransposition activity in Condensin II depleted cells led us to investigate whether CAP-D3/Condensin II regulates endogenous L1 RNA and/or protein levels. Indeed, siRNA-mediated depletion of CAP-D3 or CAP-H2 resulted in an increase in endogenous L1 ORF1 containing RNA levels as compared to cells transfected with control siRNA (Fig 2A; Decreased levels of CAP-D3 and CAP-H2 protein in these cells are shown in a later figure). Notably, the increase in L1 ORF1 containing RNA levels was also observed in non-transformed, hTERT-immortalized, CAP-D3 deficient RPE-1 cells (Fig 2B), suggesting that CAP-D3’s inhibition of L1 RNA accumulation is not restricted to cells derived from tumors. These qRT-PCR analyses measure expressed sequences containing L1 ORF1 sequence, including those present in pseudogenes or introns of genes. Therefore, to more accurately determine whether CAP-D3 depletion affects transcription/accumulation of full-length L1 RNA, we performed RNA FISH using two distinct probe sets. One probe set was directed against the 5’UTR and the second against the 3’UTR of L1 RNA. We observed a significant increase in the number of foci containing full-length L1 RNA, as denoted by colocalization of the two FISH probes, in cells depleted of CAP-D3 compared to control (Fig 2C and 2D). Furthermore, significantly more full-length L1 RNA containing foci were observed in both the nucleus and cytoplasm in cells depleted of CAP-D3, as compared to control (Fig 2E and 2F). Finally, a higher number of colocalized foci were observed in the cytoplasm of CAP-D3 deficient cell in comparison to the nucleus. Taken together, these data suggest that decreased expression of CAP-D3 results in increased transcription and may also result in increased stability of L1 mRNAs.

A number of cellular defense mechanisms are known to regulate L1 RNA levels via interaction with the L1 RNA intermediate [57, 60, 93–95]. Since our Immuno-FISH experiments demonstrated colocalization of CAP-D3 with full length L1 RNA containing foci (Fig 2C), we tested whether CAP-D3 could associate with L1 RNA. RNA-immunoprecipitation assays were performed in HT-29 cells transfected with a pLRE-*mEGFP1* L1 expression plasmid. Cross-linked, whole cell lysates were incubated with CAP-D3 antibody, total RNA was purified from unbound and bound fractions, and then used to make cDNA and qRT-PCR was performed. The amount of RNA-bound protein was calculated by dividing the RNA level present in the bound fraction of the sample by the RNA level present in the unbound fraction of the same sample so as to account for differences in total RNA between samples from different experimental replicates. Indeed, RNA immunoprecipitation (RIP) analyses showed that CAP-D3 is capable of associating with L1 RNA in both the nuclear and the cytoplasmic fractions (Fig 2G). However, our assays do not allow us to determine whether the binding is direct or indirect. As a control, similar experiments were performed following omission of reverse transcriptase, resulting in no amplification of sequence (S3 Fig).

### Condensin II binds EPRS, a novel repressor of L1 retrotransposition

**Fig 3:**
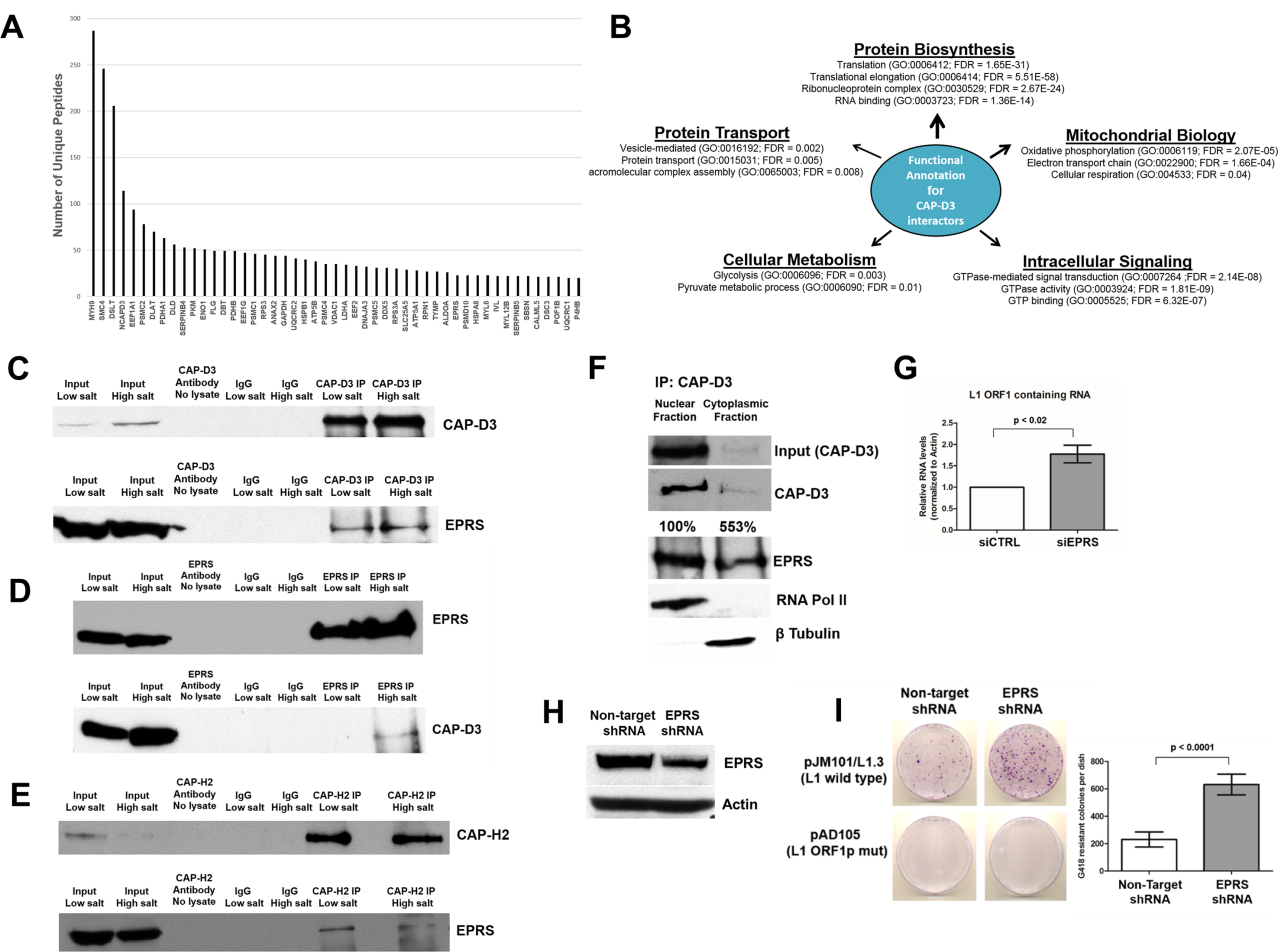
Condensin II associates with another repressor of L1 retrotransposition, EPRS. (A) The top 50 CAP-D3 binding partners predicted by immunoprecipitation/mass spectrometry ranked according to total spectral count. (B) Functional annotation of all putative CAP-D3 binding partners as determined by the Database for Annotation, Visualization, and Integrated Discovery (*DAVID*). A Benjamini corrected FDR < 0.05 was considered significant. (C-E) Immunoprecipitation of CAP-D3, CAP-H2 or EPRS in HT-29 cells under both low and high salt lysis conditions, followed by immunoblotting for the indicated proteins. Immunoprecipitations with antibody only (no lysate) and with IgG antibody were performed as controls for both lysis conditions. (F) CAP-D3 immunoprecipitations in nuclear and cytoplasmic fractions of HT-29 cells, followed by immunoblotting for EPRS or RNA polymerase II and β-tubulin to confirm isolation of nuclear and cytoplasmic fractions, respectively. Percentages indicate the amount of EPRS pulled down, compared to the amount of CAP-D3 immunoprecipitated in each fraction. (G) qRT-PCR analyses of endogenous L1 ORF1 containing RNA levels in HT-29 cells transfected with siRNA directed against EPRS, as compared to control siRNA transfected cells. (H) Immunoblotting for EPRS in HT-29 colorectal adenocarcinoma cells expressing inducible Non-Target (control) or EPRS shRNA. Actin was used as a loading control. (I) Retrotransposition assays involving wild-type (top row) or mutant/defective (bottom row) L1 elements in Non-Target or EPRS shRNA expressing cells. Crystal violet stained drug-resistant foci were quantified using ImagePro for each cell type and quantifications are shown in the chart on the right.

As CAP-D3 does not possess canonical nucleic acid binding motifs, we hypothesized that additional proteins may cooperate with CAP-D3 to bind to L1 RNAs. To better understand the mechanisms behind CAP-D3 association with L1 RNA, we immunoprecipitated CAP-D3 from HT-29 cells using high and low salt extraction buffers and analyzed co-precipitated proteins by Liquid Chromatography-Mass Spectrometry (LC-MS). A significant number of CAP-D3 peptides were identified by LC-MS (Fig 3A and S1 Table). An LC-MS-identified protein was selected as a putative CAP-D3-interacting candidate if it met the following criteria: 1) present in both low and high salt lysis conditions, 2) predicted by three or more unique peptides (peptide error rate ≤0.05, protein probability ≥0.95), and 3) not present in control immunoprecipitates. Based on these criteria, 171 putative CAP-D3 interacting proteins were identified (S1 Table). The top 50 putative CAP-D3 binding partners with the largest number of unique peptides identified are shown in Fig 3A. As expected, we identified many SMC4 and CAP-G2 peptides, other Condensin II members, as significant CAP-D3 interactors in our analyses (S1 Table). All putative CAP-D3 interactors were functionally annotated by *DAVID* gene ontology analysis (https://david.ncifcrf.gov/) [96, 97]. A number of proteins were predicted to be involved in functions ranging from mitochondrial biology to protein transport and cellular metabolism (p≤0.05) (Fig 3B). Interestingly, more than 35% of putative CAP-D3 binding partners were annotated to possess roles in RNA binding and the regulation of protein translation/ biosynthesis (Fig 3B).

One of the newly identified CAP-D3 binding partners, EPRS, is a glutamyl-prolyl tRNA synthetase known to bind RNA and recently identified as a component of the L1 RNP, suggesting that it may also regulate retrotransposition [59, 83]. Immunoprecipitation for CAP-D3 in HT-29 cells and reciprocal immunoprecipitations for EPRS in high salt lysis conditions confirmed the interaction between the two proteins (Fig 3C, Fig 3D). Excitingly, immunoprecipitation for CAP-H2 in HT-29 cells also co-precipitated EPRS, suggesting that the interaction involves the entire Condensin II complex (Fig 3E). Furthermore, Condensin II’s ability to bind EPRS likely occurs in non-transformed, primary cells, as immunoprecipitations performed using lysates from epithelial cells isolated from resected human colon tissue also show an interaction between CAP-D3 and EPRS (S4 Fig). Immunoprecipitation of CAP-D3 followed by immunoblotting for EPRS from nuclear and cytoplasmic fractions of HT-29 cells indicates that the interaction between CAP-D3 and EPRS occurs in both the nucleus and the cytoplasm of the cell (Fig 3F) and the interaction appears to occur more frequently in the cytoplasm.

While EPRS was previously identified to associate with the L1 RNP, it was not studied as a potential regulator of L1 retrotransposition. HT-29 cells transfected with EPRS siRNAs showed a significant increase in ORF1 RNA levels, as compared to control siRNA transfected cells (Fig 3G; Decreased levels of EPRS protein in these cells are shown in a later figure). L1 retrotransposition assays demonstrated significant increases in G418-resistant foci following knockdown of EPRS, suggesting that decreased EPRS expression results in increased L1 activity (Fig 3H, Fig 3I), similar to results seen in CAP-D3-deficient cells (Fig 1). Together these results indicate that Condensin II binds EPRS and, like Condensin II, EPRS acts to restrict L1 retrotransposition.

### CAP-D3 and GAIT cooperate to interact with L1 RNA

**Fig 4:**
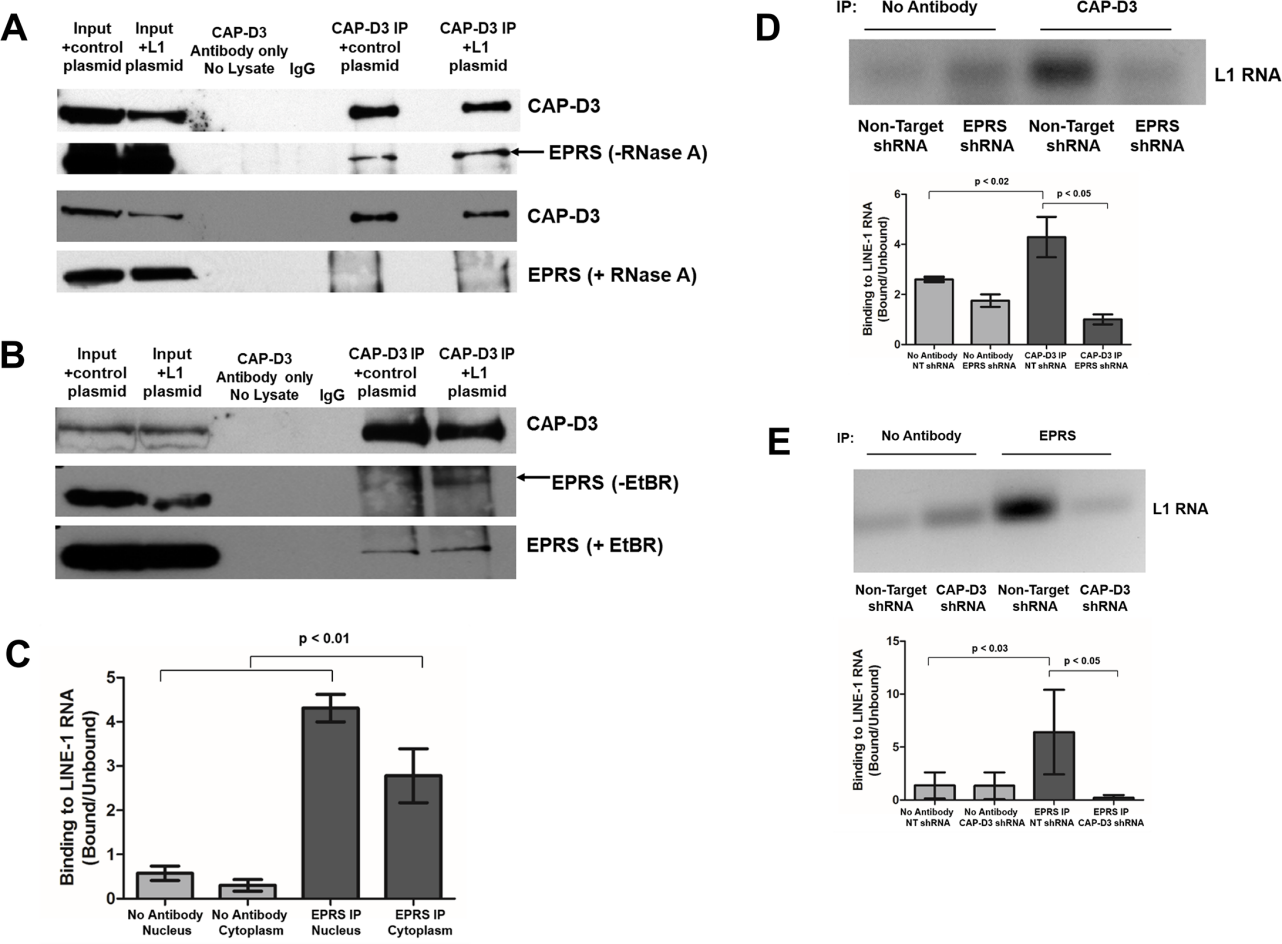
CAP-D3 and EPRS cooperate to associate with LINE-1 RNA. (A,B) CAP-D3 immunoprecipitation followed by immunoblotting for CAP-D3 or EPRS in the presence and absence of RNase A (A) or Ethidium Bromide (B) in HT-29 cells transfected with a control plasmid (pCEP4) or a plasmid expressing L1 (pJM101/L1.3) to increase active retrotransposition. (C) Quantitation of RNA-IP assays using EPRS antibody or no antibody in cells fractionated into nuclear and cytoplasmic fractions. Binding of EPRS to L1 RNA was normalized to the signal intensity in the unbound fractions for each condition (n = 3). (D) RNA-IP assays using CAP-D3 antibody or no antibody in lysates from Non-Target or EPRS shRNA expressing cells. Binding of CAP-D3 to L1 RNA in the presence and absence of EPRS was normalized to the signal intensity in the unbound fractions for each condition (n = 3). Quantitation is shown in the bottom panel. (E) RNA-IP assays using EPRS antibody or no antibody in lysates from Non-Target or CAP-D3 shRNA expressing cells. Binding of EPRS to L1 RNA in the presence and absence of CAP-D3 shRNA was normalized to the signal intensity in the unbound fractions for each condition (n = 3). Quantitation is shown in the bottom panel. P-values were calculated with a student t-test.

We hypothesized that, as a means to repress L1 retrotransposition, CAP-D3 and EPRS might cooperatively interact with L1 RNAs. In support of this idea, immunoprecipitation of CAP-D3 conducted in the presence of RNase A disrupted the association between CAP-D3 and EPRS (Fig 4A). However, binding of EPRS to CAP-D3 remained largely unchanged in lysates treated with ethidium bromide to disrupt protein interactions dependent on the presence of DNA (Fig 4B). Interestingly, immunoprecipitation for CAP-D3 in cells transfected with L1 compared to cells transfected with an empty vector showed a small, but consistently visible increase in the association between CAP-D3 and EPRS, lending further support to the idea that increased levels of L1 RNA invokes cooperation between the two proteins (Fig 4A, Fig 4B black arrow).

RNA-immunoprecipitation assays revealed that EPRS can associate with L1 RNA in both the nuclear and cytoplasmic fractions (Fig 4C). Excitingly, RNA-immunoprecipitation experiments performed in Non-Target, CAP-D3, or EPRS shRNA expressing HT-29 cells transfected with the pLRE-*mEGFP1* L1 expression plasmid demonstrated that knockdown of EPRS or CAP-D3 by shRNA, results in significant decreases in binding of the other protein to L1 RNA, suggesting that the interaction of CAP-D3 and EPRS with L1 RNAs occurs in a co-dependent manner (Fig 4D and 4E). Together, these results illustrate cooperation between CAP-D3/Condensin II and EPRS and suggest they may work together to repress L1 mobilization through their co-dependent interaction with L1 RNAs.

### CAP-D3/Condensin II bind to and promote the association of GAIT complex subunits

EPRS is a component of several complexes with diverse functions. In an effort to understand which of these functions might be important for its ability to bind to CAP-D3 and to repress L1 retrotransposition, we first addressed whether EPRS might bind to CAP-D3/Condensin II as part of the Multi-Synthetase Complex (MSC). The MSC is composed of EPRS and seven additional enzymes and plays important roles in protein synthesis [98]. EPRS dissociates from the MSC following phosphorylation at two distinct serine residues, allowing it to form complexes with other proteins [80]. Interestingly, immunoprecipitation of CAP-D3 from nuclear and cytoplasmic fractions revealed binding to phosphorylated EPRS^Ser886^ in both fractions (S5 Fig). Furthermore, siRNA-mediated depletion of another component of the MSC, the tRNA synthetase FARSA, resulted in no changes to ORF1 RNA or protein levels (S6 Fig). Therefore, these data suggest that EPRS most likely does not require the other components of the MSC to bind CAP-D3 or repress L1.

**Fig 5:**
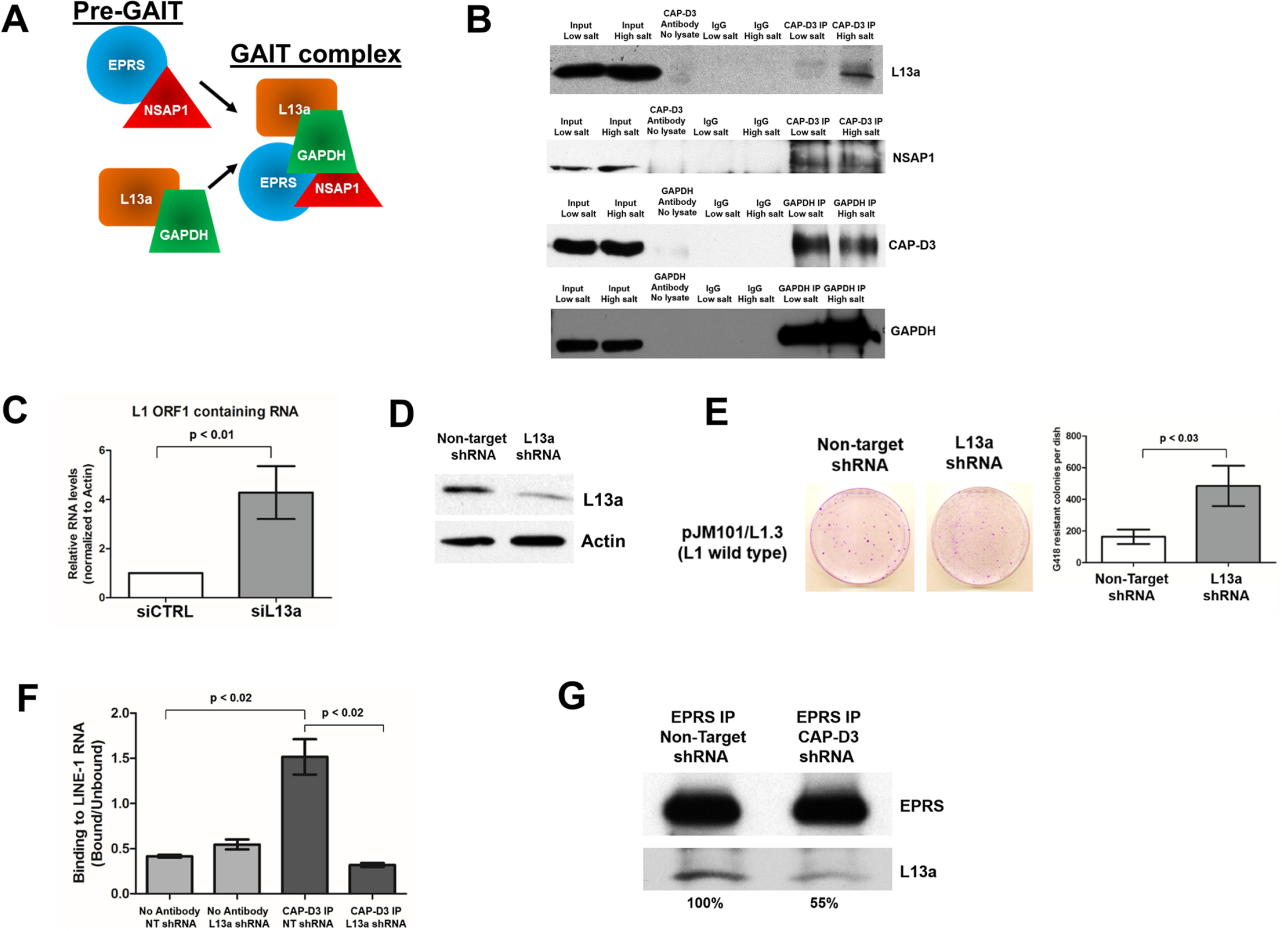
CAP-D3 binds GAIT complex members which also repress retrotransposition in colon epithelial cells. **(**A) Diagram of the Pre-GAIT complex (EPRS and NSAP1) associating with L13a and GAPDH to form the GAIT complex. (B) CAP-D3 immunoprecipitation and immunoblotting for GAIT complex members L13a (top row) or NSAP1 (second row) under both low and high salt lysis conditions. Immunoprecipitation for GAIT subunit GAPDH followed by immunoblotting for CAP-D3 or GAPDH under both low and high salt lysis conditions is shown in the third and fourth rows. All immunoprecipitations were performed with antibody only (no lysate) and with IgG antibody as controls for both lysis conditions. (C) qRT-PCR for endogenous L1 ORF1 containing RNA levels in HT-29 cells transfected with siRNA directed against L13a, as compared to control siRNA transfected cells. (D) Immunoblotting for L13a in HT-29 colorectal adenocarcinoma cells expressing inducible Non-Target (control) or L13a shRNA. Actin was used as a loading control. (E) Retrotransposition assays involving wild-type L1 ORF1p elements in Non-Target or L13a shRNA expressing cells. Crystal violet stained drug-resistant foci were quantified using ImagePro for each cell type and quantitations are shown in the chart on the right. (F) Quantitation of RNA-IP assays using CAP-D3 antibody or no antibody, as a control, in lysates from Non-Target or L13a shRNA expressing cells. Binding of CAP-D3 to L1 RNA in the presence and absence of L13a shRNA was normalized to the signal intensity in the unbound fractions for each condition (n = 2). (G) EPRS immunoprecipitation and immunoblotting for L13a in Non-Target or CAP-D3 shRNA expressing HT-29 cells. Percentages indicate level of L13a binding to EPRS following immunoprecipitation. P-values were calculated with a student t-test.

In myeloid cells, EPRS has been shown to function as part of a larger protein complex, the Gamma Interferon-Activated Inhibitor of Translation (GAIT) complex. GAIT is composed of EPRS, the ribosomal protein L13a, GAPDH, and NSAP1 (Fig 5A). In this context, GAIT has been shown to repress translation of select transcripts in response to IFN-γ [99]. Although HT-29 cells do not express IFN-γ (S7 Fig and [89]), we tested whether a GAIT-like complex might act independently of IFN-γ to repress L1 RNA translation in coordination with CAP-D3. This idea was supported by the fact that the LC-MS/MS analysis identified L13a and GAPDH as putative CAP-D3 binding partners (Fig 3). We confirmed the interactions between L13a, NSAP1, GAPDH and CAP-D3 by immunoprecipitation/immunoblot analyses in HT-29 cells (Fig 5B).

L13a is an essential member of the GAIT complex. Following its association with the pre-GAIT complex (EPRS and NSAP1) and binding to the target mRNA, L13a will bind to eIF4G, a member of the translation initiation complex, and prevent translation by blocking assembly of the ribosome onto the RNA platform [82, 100, 101]. To determine whether L13a regulates endogenous L1 expression, we transfected cells with L13a siRNA and performed qRT-PCR for L1 ORF1 containing RNAs. Similar to knockdown of CAP-D3 and EPRS, decreases in L13a expression resulted in significant increases in endogenous ORF1 RNA levels in HT-29 cells (Fig 5C). Additionally, results of L1 retrotransposition assays in HT-29 cells expressing L13a shRNA demonstrated significant increases in G418-resistant foci suggesting that L13a does, in fact, act to repress L1 retrotransposition (Fig 5D, Fig 5E). Interestingly, the G418-resistant foci in the L13a shRNA expressing cells were often very small compared to those in the Non-Target shRNA expressing cells, potentially due to the possibility that L13a is essential for cell growth in this cell type. To determine whether additional GAIT subunits (aside from EPRS) were necessary for the association between CAP-D3 and L1 RNA, we performed RNA immunoprecipitation assays for CAP-D3 in cells expressing L13a or Non-target shRNA. Similar to depletion of EPRS, knockdown of L13a resulted in significantly decreased association of CAP-D3 with L1 RNA (Fig 5F).

Unexpectedly, immunoprecipitation studies in HT-29 cells also showed that EPRS and L13a association is decreased following CAP-D3 depletion (Fig 5G). Given that the Pre-GAIT complex, containing EPRS and NSAP1, recruits L13a to promote formation of the active GAIT complex [100, 102], these results suggest that CAP-D3 may be important for converting the inactive Pre-GAIT complex to the active GAIT complex in colon epithelial cells. We investigated whether CAP-D3 regulates expression of EPRS and/or L13a, but saw no changes in protein levels upon CAP-D3 depletion, suggesting that the increases in retrotransposition observed in CAP-D3 deficient cells are not indirectly due to decreasing levels of essential GAIT complex components (S8 Fig).

### L1 translation is restricted by Condensin II and GAIT

**Fig 6:**
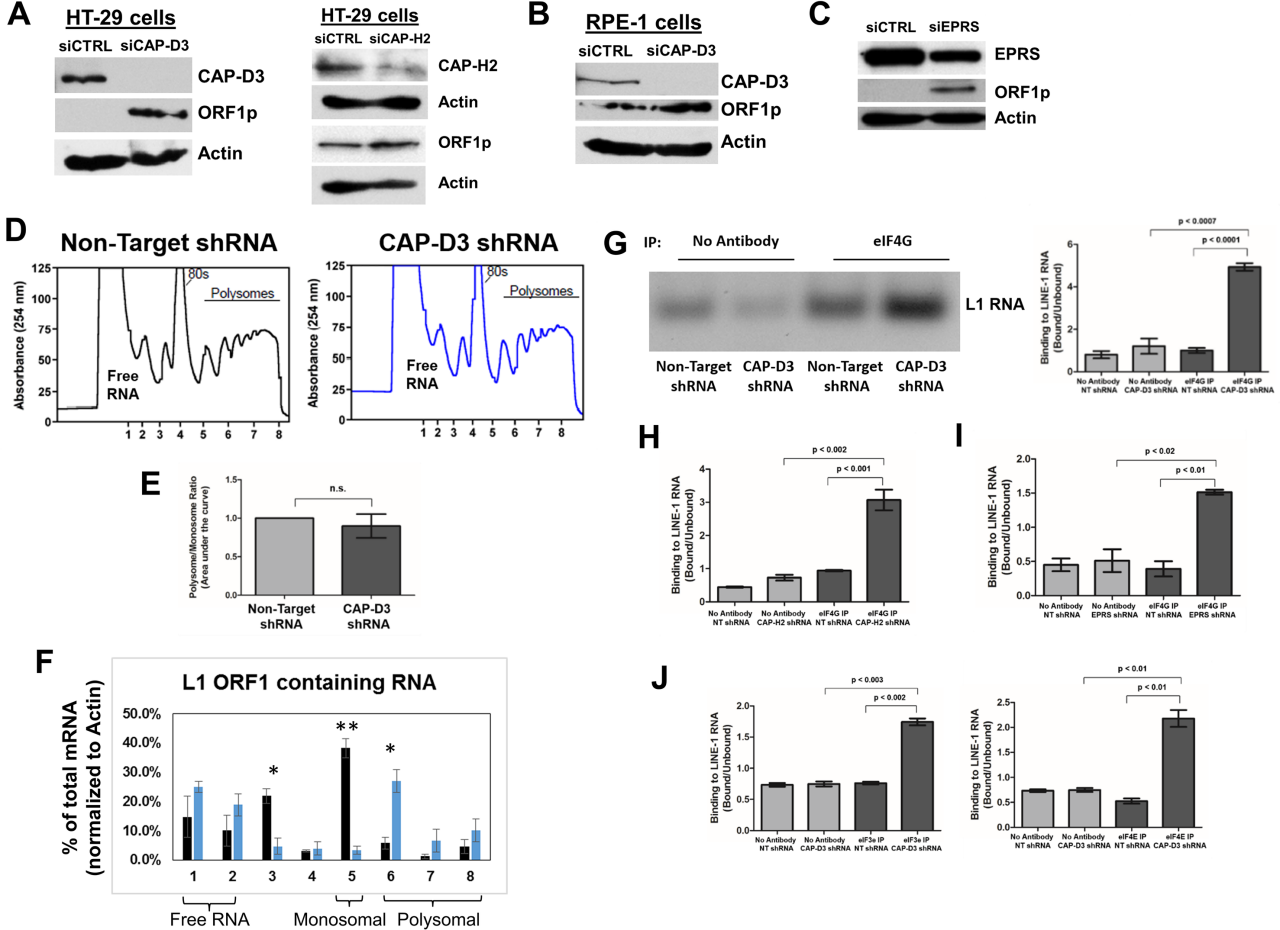
Condensin II and GAIT inhibit translation initiation and eIF4G association with the LINE-1 RNA. (A, B) Immunoblotting analysis of endogenous L1 ORF1 protein in CAP-D3 or CAP-H2 siRNA transfected HT-29 (A) and CAP-D3 siRNA transfected RPE-1 cells (B) compared to control siRNA transfected cells. Actin was used as a loading control. n≥3 for each experiment. (C) Immunoblotting analysis of endogenous L1 ORF1 protein in EPRS siRNA (left panel) transfected HT-29 cells compared to control siRNA transfected cells. Actin was used as a loading control. n = 3. (D) Polysome profiles in Non-Target and CAP-D3 shRNA expressing cells (charts shown are representative of the experiments performed). Fractions containing Free RNA, Monosome-associated and Polysome-associated RNA are labelled. (E) Ratio of the area under the polysomal (P) to monosomal (M) peaks is shown (P:M). (F) qRT-PCR analysis of L1 ORF1 containing RNA levels across all polysome fractions, calculated as percentages of total RNA for the sample. Results were normalized to Actin levels for each fraction, and are presented as the average of two independent experiments. Free RNA fractions, the monosome-associated RNA fractions, and the polysome-associated RNA fractions are labeled underneath the chart. P-values were calculated with a student t-test. * p<0.05, ** p<0.005. (G) RNA-IP assays using eIF4G antibody or no antibody in lysates from Non-Target or CAP-D3 shRNA expressing cells. Binding of eIF4G to L1 RNA in the presence and absence of CAP-D3 shRNA was normalized to the signal intensity in the unbound fractions for each condition (n = 3). (H,I) Quantitation of RNA-IP assays using eIF4G antibody or no antibody in lysates expressing Non-Target, CAP-H2 (H) or EPRS (I) shRNAs. Binding of eIF4G to L1 RNA was normalized to the signal intensity in the unbound fractions for each condition (n = 2). P-values were calculated with a student t-test. (J) Quantitation of RNA-IP assays using eIF3e (left panel), eIF4E (right panel), or no antibody in lysates expressing Non-Target or CAP-D3 shRNAs. Binding of eIF3e and 4E to L1 RNA was normalized to the signal intensity in the unbound fractions for each condition (n = 2). P-values were calculated with a student t-test. A *p*-value < 0.05 was considered statistically significant.

The GAIT complex binds mRNAs circularized by simultaneous interactions of poly(A)-binding protein with the translation initiation factor eIF4G and the poly(A) tail [82]. GAIT then represses translation through binding of L13a to eIF4G which blocks recruitment of the eIF3-containing 43S ribosomal subunit, thus prohibiting formation of the translation initiation complex at the 5’UTR of the targeted transcript [82]. Since our data demonstrated binding between Condensin II, GAIT and L1 RNA, we hypothesized that Condensin II might co-opt the translation inhibiting functions of GAIT to repress L1 translation. To test this, we first immunoblotted for L1 ORF1p in cells depleted of CAP-D3. In accordance with qRT-PCR and FISH results which demonstrated increased L1 mRNA levels (Fig 2A), immunoblot analyses also showed increased L1 protein levels, both in transformed and untransformed cells (Fig 6A, Fig 6B, S9A Fig, S9B Fig). This was also true in cells depleted of EPRS and L13a (Fig 6C, S9C Fig).

To better assess whether CAP-D3/Condensin II can influence the translation of L1 RNA, we performed three, independent polysome profiling analyses. In these assays, cytoplasmic lysates from Non-Target or CAP-D3 shRNA expressing cells were fractionated over a sucrose gradient. Total RNA was extracted from each of the 8 fractions collected, and levels of L1 RNAs present among the free RNA, monosome-associated, and polysome-associated RNA-containing fractions were assessed by qRT-PCR using primers directed against L1 ORF1. The ratio of the area under the polysomal (P) to monosomal (M) peaks was determined (P:M), and indicated that knockdown of CAP-D3 does not alter global translation compared to Non-Target shRNA expressing cells (Fig 3D, Fig 3E). Strikingly, qRT-PCR results showed decreases in monosome-associated ORF1 containing RNAs and concomitant increases in polysome-associated ORF1 containing RNAs following knockdown of CAP-D3, (Fig 6F). This data suggests that loss of CAP-D3 may result in an increase in actively translating L1 RNAs, compared to Non-Target shRNA expressing control cells.

Interestingly, RNA-IP results showed that eIF4G binding to L1 RNAs significantly increases in the absence of CAP-D3 (Fig 6G), CAP-H2 (Fig 6H), or EPRS (Fig 6I). These results were also verified in experiments using primers directed against EGFP sequence and immunoprecipitates from cells transfected with an EGFP-tagged L1 construct (S10A–S10C Fig). Similar to eIF4G, we observed that loss of CAP-D3 results in a significant increase in binding of additional translation initiation factors, eIF3e and eIF4E, to the L1 RNA (Fig 6J). As a control, GAPDH levels were monitored and no significant changes in eIF4G binding to GAPDH RNAs were observed following CAP-D3 knockdown (S10D Fig). Notably, depletion of CAP-D3 does not affect protein levels of all GAIT targets in epithelial cells, since protein levels of the GAIT target, ZIPK, remained largely unchanged (S11 Fig). However, as ZIPK was identified as a GAIT target in monocytes, it is possible that epithelial cell-specific GAIT targets a completely different set of mRNAs. Taken together, these data support a cooperative role for the Condensin II and GAIT complexes in the inhibition of L1 protein translation in epithelial cells.

## Discussion

Repression of L1 is important for maintaining genome stability. Our data indicates that CAP-D3/Condensin II inhibits retrotransposition in human primary and transformed epithelial cells at multiple levels. qRT-PCR data and FISH experiments indicate that CAP-D3/Condensin II represses transcription of L1 RNAs (Fig 2). Immunoblotting analyses of L1 ORF1p, as well as polysome profiling experiments analyzing L1 mRNAs suggest that CAP-D3/Condensin II also represses L1 protein translation (Fig 6). In this manuscript, we have identified a novel interaction between Condensin II subunits and members of the GAIT complex and demonstrated the cooperative potential of the two multi-subunit protein complexes in maintaining L1 repression in somatic cells. CAP-D3/Condensin II directly or indirectly associates with L1 RNA and promotes GAIT complex assembly. CAP-D3 requires the association with GAIT to interact with L1 RNAs and, together, the two complexes prevent binding of translation initiation factors to L1 RNAs. Combined, these results uncover one of the potential mechanisms used by CAP-D3/Condensin II and GAIT to repress L1 activity in human epithelial cells.

Canonical GAIT complex assembly is initiated following IFN-γ treatment as a mechanism to inhibit translation of RNAs encoding inflammatory proteins [102]. Our studies indicate that the ability of the Condensin II and GAIT complexes to inhibit L1 retrotransposition is independent of IFN-γ stimulation. While previous reports have shown that Condensin II subunits can localize to the cytoplasm of the cell [65, 69, 103], little is known about the cytoplasmic roles of Condensin II, and our data suggests that regulation of translation could be one of these functions. While global changes in translation following CAP-D3 knockdown were not observed in polysome profiling analyses (Fig 6E), it is possible that CAP-D3 could be involved in regulating the translation of a small subset of RNAs in addition to L1 ORF1p. Our mass spectrometry data revealed that, in addition to GAIT complex members, CAP-D3 interacts with many other proteins involved in the process of translation, including ribosomal proteins and translation elongation factors. While we have not validated these proteins as novel CAP-D3 binding partners, these findings lend support to the hypothesis that CAP-D3/Condensin II might be capable of regulating translation of many RNAs within the cell. Furthermore, it will be interesting to determine whether CAP-D3/Condensin II responds to IFN-γ in other cell types, such as epithelial cells, to regulate translation of specific messages, including L1, in coordination with GAIT. It has been reported that increased L1 activity does in fact lead to IFN-γ expression in fibroblasts [104]. Therefore, the question of whether Condensin II/GAIT can act downstream of IFN-γ to repress L1 becomes interesting and relevant.

Another question that arises from the work presented here is whether the DNA organizing functions of Condensin II may be important for its ability to repress L1 translation. Many architectural DNA-binding proteins such as Condensin II, which organize DNA at the global and local levels to create topologically-associating domains, have been shown to regulate gene expression [78, 105, 106]. CAP-D3 organizes retrotransposon loci in *Drosophila* and regulates repressive histone marks to control RNA levels [62]. In *Drosophila*, it has been described that gypsy retrotransposon sequences with insulator activity can cause loci to move to the nuclear periphery [107]. Additionally, in *Drosophila*, lamin depletion results in a reduction of heterochromatin and a corresponding increase in retrotransposon expression and DNA damage, suggesting the importance of the nuclear lamina in maintaining genome integrity by repressing retrotransposons [108]. Our data demonstrates that CAP-D3 associates with EPRS in both the nuclear and cytoplasmic fractions, but the exact subcellular locations/organelles where they bind L1 RNAs are currently unknown. One possible model that would incorporate both the ability of CAP-D3/Condensin II to repress L1 RNA translation and to organize DNA would be that Condensin II might organize L1-containing loci at the nuclear periphery in repressive domains. If transcription does occur, then Condensin II and EPRS could bind L1 RNA and, following transport into the cytoplasm, block eIF4G binding and translation initiation with the help of the entire GAIT complex. A more thorough investigation of how CAP-D3/Condensin II regulates the positioning of retrotransposons in human cells, and whether it requires EPRS, is necessary.

Excitingly, our data demonstrates that CAP-D3 can associate with RNA although future studies are needed to determine whether this association is direct or indirect. Proteins known to associate with and/or modify chromatin have recently been shown to widely interact with both coding and noncoding RNA transcripts [109]. Interestingly, chromatin-associated proteins have been shown to bind to specific transposable element (TE) families through evolutionarily conserved sequence motifs, which may be functionally important for their regulation[109]. The ability of the GAIT complex to inhibit protein translation is dependent on a specific element in the 3’UTR (or less frequently in the 5’UTR) of transcripts, which has been termed the GAIT element [reviewed in [102]]. The secondary structure of the GAIT element consists of a stem-loop with an asymmetric internal bulge. Analysis of the L1 3’UTR and 5’ UTR using RNAFold (http://rna.tbi.univie.ac.at) [110], identified two potential GAIT-like elements in the 3’UTR of L1.3 RNA (black arrows, S12A Fig). One of these elements is not present in the secondary structure of the L1.3 sequence present in pJM101 (used throughout this work), as it is disrupted by the neomycin cassette (S12B Fig). Intriguingly, analysis of the 3’UTR of two *Drosophila* retrotransposons using RNAFold, also identified potential GAIT-like elements in both the LTR-containing retrotransposon, mdg1 and the LINE-like retrotransposon, X-element (S12C Fig). We previously observed that CAP-D3 represses RNA levels and retrotransposition of these retrotransposons in *Drosophila* [62]. It will be interesting, in future studies, to determine whether any of these elements are important for the ability of Condensin II and GAIT to repress L1 retrotransposition.

The novel role for Condensin II in repressing L1 retrotransposition could be important for understanding the effects of Condensin II depletion or loss of function on the development of human disease. Retrotransposon activity is linked to the progression of many life-threatening cancers, including lung, colon, and breast cancer [3, 14–19, 111, 112]. Interestingly, CAP-D3 mutations have been found in different types of somatic cancers and mutations in all three non-SMC proteins (CAP-H2, CAP-D3 and CAP-G2) occur at significant levels in lung squamous cell carcinoma and lung adenocarcinoma [113]. Additionally, in subtype-1 prostate tumors, CAP-D3 expression was associated with decreased tumor recurrence after radical prostatectomy, independent of pathologic tumor stage [114]. Recently, Woodward and colleagues observed that mice inheriting missense mutations in CAP-H2 develop T-cell lymphoma with extreme aneuploidy [84]. Additionally, loss of function mutations in both of the structural maintenance of chromosomes (SMC) subunits have been found in Pyothorax-associated lymphoma (PAL) tumor samples exhibiting inaccurate chromosome segregation and abnormal chromosome length [115]. Our lab has shown that CAP-D3 protein levels are reduced in patients with active Ulcerative Colitis (UC), an inflammatory bowel disease (IBD) that involves chronic inflammation [89]. UC is also predisposing for colitis-associated colon cancer [116]. This is particularly relevant to our findings because loss of CAP-D3 in the inflammatory bowel context could result in more active L1 retrotransposition, thus contributing to genome instability and predisposition for tumor development. However, this idea needs to be thoroughly tested in a large cohort of patient cells. The data presented here uncover a new potential mechanism of L1 repression by two multi-functional protein complexes. Our results not only improve our knowledge of the control of retrotransposition, but also identify new proteins and pathways that could act as potential therapeutic targets to prevent tumor development.

## Materials and methods

### Ethics statement

Intestinal epilthelial cells were isolated from resected patient tissues in accordance with the Cleveland Clinic Institutional Review Board protocol #05–205.

### Cell lines

All mammalian cell culture lines were obtained from the American Type Culture Collection (ATCC). HT-29 cells (HTB-38) were cultured in RPMI 1640 media with 10% fetal bovine serum (FBS; Life Technologies), and 1% penicillin/streptomycin. hTERT-RPE-1 cells (CRL-4000) were cultured in DMEM-F12 media with 10% FBS, and 1% penicillin/streptomycin. Caco-2 cells (HTB-37) were cultured in DMEM media, with sodium pyruvate, 10% FBS, and 1% penicillin/streptomycin.

### Epithelial cell isolation from resected patient colon tissues

Epithelial cells were isolated from resected colonic tissue of six patients (five female and one male) with diverticulitis, ranging in age from 54–80 (Cleveland Clinic Tissue Procurement Service; Institutional Review Board protocol #05–205). Epithelial cells were isolated as previously described [89].

### Inducible small hairpin RNA cell lines

Lentivirus transduction was performed in HT-29 cells using custom viral particles produced with the pLKO-puro-IPTG-3xLacO vector (Sigma-Aldrich). As described previously, in the absence of IPTG (isopropyl-β-D-thio-galactoside), an analogue of lactose, LacI binds to LacO preventing expression of the shRNA[89]. When IPTG is present, the allosteric LacI repressor changes conformation, releasing itself from LacO modified human U6 promoter, and subsequently allows expression of the shRNA. Cells were plated in a 12-well plate in 1mL of media/well and incubated at 37°C overnight. Growth medium was prepared with 8μg/ml of polybrene and added to each well the following day. Non-target and CAP-D3 shRNA expressing HT-29 and Caco2 cell lines were previously described [89]. To manufacture the additional HT-29 cell lines, CAP-H2, EPRS, or L13a shRNA lentiviral solution (50μl, 1000U/μl) was added to the cells, gently mixed and incubated for 8 hours. After incubation, the polybrene containing media was changed to normal growth media. Cells were selected with puromycin (12μl/ml) 3 days after transduction. Stable clones were validated by qPCR and immunoblotting for decreased protein expression.

### Plasmid constructs

***pCEP4*:** A mammalian expression vector (Life Technologies) used to construct some of the L1 plasmids (as indicated below) and contains a cytomegalovirus (CMV) promoter and an SV40 polyadenylation signal. The plasmid backbone also contains a hygromycin resistance gene (HYG) and the Epstein Barr Virus Nuclear Antigen-1 gene (EBNA-1).

***pJM101/L1*.*3*:** A pCEP4-based plasmid that contains an active human L1 (L1.3) equipped with a *mneoI* retrotransposition indicator cassette [22, 90, 91].

***pAD105*:** An inactive allele of L1.3, which contains the RR_261–262_AA mutation in the ORF1p C-terminal domain [22, 35].

***pLRE3-mEGFPI*:** A pCEP4-based plasmid that contains an active human L1 (LRE3) equipped with a *mEGFPI* retrotransposition indicator cassette [117]. The pCEP4 backbone was modified to contain a puromycin resistance (PURO) gene in place of the HYG. The CMV promoter also was deleted from the vector; thus, L1 expression is only driven by its native 5′-UTR [117].

### Antibodies

The following primary antibodies utilized throughout this study for immunoblotting or immunoprecipitation: CAP-D3 (Bethyl Laboratories), CAP-H2 (Bethyl Laboratories), EPRS (Bethyl Laboratories), L13a (Cell Signaling Technology), NSAP1 (AnaSpec Inc), GAPDH (Santa Cruz), eIF4G (Santa Cruz), eIF3e (Abcam), eIF4E (Abcam), IFN-γ (Biosource), Actin (Millipore), RNA Polymerase II (Abcam), β-tubulin (Cell Signaling Technology), Normal Rabbit IgG (Millipore). Purified polyclonal α-ORF1p was generated by OpenBiosystems and characterized by the laboratory of John Moran (University of Michigan School of Medicine[57]). EPRS WHEP-linker antibody was previously described [118].

### siRNA-mediated knockdown of cellular proteins

Lipofectamine 2000 (Invitrogen) was used according to the manufacturer’s direction to transfect Non-Targeting (40nM; D-001206-13-05, siGENOME Non-Targeting siRNA pool), CAP-D3 (20nM; M-026539-01-0005, siGENOME Human NCAPD3 (23310) siRNA–SMARTpool), CAP-H2 (40nM; D-016186-03-0005, siGENOME Human NCAPH2 (29781) siRNA–Individual), EPRS (40nM; s4767 and s4768, Silencer Select Human glutamyl-prolyl-tRNA synthetase), or L13a (10nM; s198709, Silencer Select Human L13a) specific siRNAs into HT-29 or RPE-1 cells. Briefly, ~8x10^4^ cells were plated in 12-well plates and transfected the following day. Twenty-four to 72 hours following transfection, depending on the siRNA being utilized, protein and/or RNA were harvested to assess knockdown efficiency.

### Cell-culture based retrotransposition assay

Retrotransposition assays were performed as described previously with minor modifications [22, 60, 119]. Briefly, for G418-resistance–based retrotransposition assays, HT-29 or Caco2 cells (∼8x10^4^ per well) were seeded into two sets of six-well plates. The cells were treated with IPTG for 48 hours (unless otherwise noted) to induce shRNA expression. The cells were then co-transfected with 1μg of the indicated L1 expression plasmid and 1μg of an empty vector (pFLAG-CMV-2) using 6μl of the FuGENE 6 transfection reagent (Promega) per well. Seventy-two hours after transfection, the cells were collected from one set of plates and the pellets were frozen at -80°C for future analysis. Cells from the other set of plates were trypsinized and resuspended in complete RPMI or DMEM medium supplemented with G418 (600μg/ml) (Life Technologies). Cells from each well were split into three 10-cm tissue culture dishes, generating triplicate cultures. After 10 days of G418 selection for HT-29 cells and 15 days of drug selection for Caco2 cells, the remaining cells were treated with 10% neutral buffered formaldehyde for 5 minutes to fix them to tissue culture plates and then were stained with 0.05% crystal violet for 30 minutes to facilitate their visualization. The dishes were washed with 1X PBS and imaged with a ChemiDoc XRS+ (Bio-Rad). The images were analyzed using ImagePro Plus 7.0 software. For one 10-cm dish, a circular area of interest (AOI), which delimits the population of pixels in the image was made. The same AOI was used as a template for each subsequent image analyzed. The number of drug-resistant foci was determined within the AOI and recorded for statistical analysis.

### Immunoblotting

Total protein lysates were prepared from cells after washing the cells twice with cold 1X PBS. The cells were scraped into PBS and pelleted. The cell pellet was then resuspended in RIPA lysis buffer (300mM NaCl, 50mM Tris pH 7.5, 1mM EDTA, 0.1% Triton, 10% glycerol, 1mM DTT, 1X protease inhibitor cocktail) and incubated on ice for 30 minutes. The lysate was cleared by centrifugation and protein quantified by Bradford assay (Bio-Rad). Western blots used 50–100μg of lysate. Blot images were developed with SuperSignal West Pico Chemiluminescent Substrate (Thermo Fisher) and images captured on a Protec Ecomax X-Ray Film Processor.

### RNA FISH and immunofluorescence microscopy

HT-29 cells were plated on 18mm round coverglass in a 6-well plate then transfected with 20nM of control and CAP-D3 siRNAs in triplicate using Lipofectamine 2000. All downstream steps were performed under RNAse free conditions according to the Stellaris RNA FISH protocol (LGC BioSearch Technologies). Forty-eight hours following transfection, the coverslips were washed with 1X PBS, then fixed in 3.7% formaldehyde at room temperature for 10 minutes. The coverslips were washed twice with 1X PBS. To permeabilize cells, cells were immersed in 0.1% Triton X-100 in 1X PBS for 5 minutes at room temperature. For sequential CAP-D3 IF and FISH, cells were washed once with 1X PBS and incubated overnight in primary antibody diluted in 1X PBS at 4°C. Cells were then washed three times in PBS for 10 minutes at room temperature and incubated for 1 hour at room temperature with the fluorescently-labeled secondary antibody diluted in 1X PBS. This was followed by three 10 minute washes in 1X PBS. The coverslips were fixed for a second time in 3.7% formaldehyde for 10 minutes and then washed twice with 1X PBS. Wash Buffer A was added to the coverglass and incubated at room temperature for 5 minutes. The coverglass was transferred to a humid chamber and placed cells side down onto parafilm where 100μl of hybridization buffer (Stellaris hybridization buffer and deionized formamide), containing both a Custom L1-5’UTR Probe Set (cat. no. SMF-1065-5) and a probe set directed to all human L1RE1 (cat. no. VSMF-2982-5), was previously dispensed. The humid chamber was sealed with parafilm and incubated overnight at 37°C. The following day, the coverglass was transferred to a fresh 12-well plate with wash buffer A and incubated in the dark at 37°C for 30 minutes. Five ng/mL of DAPI in Wash Buffer A was applied to the cells and incubated in the dark at 37°C for 30 minutes. The coverglass was then washed with Wash Buffer B for 5 minutes. The coverglass was mounted using SlowFade Gold antifade mounting reagent (Life Technologies) onto a microscope slide. For experiments where IF was not performed in conjunction with FISH, the immunostaining steps were eliminated while the hybridization of the FISH probe remained the same. To obtain images, the slides were visually scanned and representative images were captured using a Leica SP5 confocal/multi-photon microscope (63x objective) that was purchased with partial funding from National Institutes of Health SIG Grant 1S10RR026820-01.

### Cell fractionation and protein isolation

For isolation of nuclear and cytoplasmic fractions, cells were grown to semi-confluency then washed twice with cold, sterile 1X PBS. Cells were harvested into 1mL of PBS and pelleted. The cell pellet was resuspended in hypotonic buffer (20mM Tris pH 8.0, 4mM MgCl_2_, 6mM CaCl_2_, 500μM DTT) and incubated on ice for 5 minutes. The sample was dounce homogenized following the addition of dounce lysis buffer (0.6M sucrose, 0.2% NP-40, 500μM DTT). The nuclei were pelleted and the resulting supernatant (cytoplasmic protein fraction) reserved for analysis. The nuclei pellet was resuspended in glycerol buffer (50mM Tris pH 8.3, 5mM MgCl_2_, 100μM EDTA, 40% glycerol) and pelleted. The pellet was then washed in 1X PBS. Finally, the nuclei pellet was resuspended in RIPA lysis buffer and sonicated for 2 seconds at level 5 (50% power) with a microtip sonicator. The sample was incubated on ice for 30 minutes and cleared by centrifugation. The resulting supernatant (nuclear protein fraction) was utilized for analysis by immunoprecipitation with the cytoplasmic fraction, following protein quantification by a Bradford assay (Bio-Rad).

### Immunoprecipitation

Cells were grown to semi-confluency, followed by whole cell lysate preparation by dounce homogenization. Cells were lysed in low (150mM NaCl, 50mM Tris pH 7.5, 1mM EDTA, 0.1% Triton, 10% glycerol, 1mM DTT, 1X protease inhibitor cocktail) and high salt (300mM NaCl, 50mM Tris pH 7.5, 1mM EDTA, 0.1% Triton, 10% glycerol, 1mM DTT, 1X protease inhibitor cocktail) containing lysis buffers. Treatment of samples with 10μg/mL RNase A (Affymetrix) was in the absence of RNase inhibitors. Treatment with 50μg/mL of ethidium bromide was also performed. Immunoprecipitations were performed using 50μl of Protein A Dynabeads (Invitrogen). Beads were washed twice with PBS/BSA solution (1X PBS, 0.2% sodium azide, 0.5% BSA), followed by blocking for 30 minutes with end-over-end rocking at room temperature. Antibodies specific to CAP-D3 (4μg), EPRS (6μg), GAPDH (4μg), eIF4G (6μg) or Normal Rabbit IgG (1μg; EMD Milipore, Billerica, MA) were incubated with the beads for 4 hours at room temperature rocking end-over-end. Finally, the beads were washed twice, for 5 minutes each with PBS/BSA at 4°C. The cleared lysate (0.5-1mg) was added to the beads and incubated overnight at 4°C. The following day, the beads were washed 4 times with 1X PBS, rocking end-over-end at 4°C. Following the final wash, the beads were suspended in 30μl of Laemmli buffer with β-Mercaptoethanol and boiled for 5 minutes. Immuno blots used 5% of IP lysate for the input lanes. Blot images were developed with SuperSignal West Pico Chemiluminescent Substrate and images captured on a Protec Ecomax X-Ray Film Processor.

### Mass spectrometry

Following immunoprecipitation, the collected supernatant was loaded on an SDS-polyacrylamide gel and stained with GelCode Blue Stain Reagent according to the manufacturer’s direction (Thermo Fisher). Mass spectrometry (MS) sequencing and database analyses was performed by the Cleveland Clinic Lerner Research Institute Proteomics Core. For the protein digestion, the bands were cut from the gel as closely as possible, washed/destained in 50% ethanol, 5% acetic acid and then dehydrated in acetonitrile. The bands were then reduced with DTT and alkylated with iodoacetamide prior to the in-gel digestion. All bands were digested in-gel, by adding 5μL of 10ng/μL trypsin in 50mM ammonium bicarbonate and incubating overnight at room temperature to achieve complete digestion. The peptides that were formed were extracted from the polyacrylamide in two aliquots of 30μL 50% acetonitrile with 5% formic acid. These extracts were combined and evaporated to <10 μL in Speedvac and then resuspended in 1% acetic acid to make up a final volume of ~30 μL for Liquid Chromatography (LC)-MS analysis. The LC-MS system used was a Finnigan LTQ-Obitrap Elite hybrid mass spectrometer system. The HPLC column used was a Dionex 15cm x 75μm id Acclaim Pepmap C18, 2μm, 100 Å reversed phase capillary chromatography column. Five microliters volumes of the extract were injected and the peptides eluted from the column by an acetonitrile/0.1% formic acid gradient at a flow rate of 0.25μL/min were introduced into the source of the mass spectrometer on-line. The microelectrospray ion source is operated at 2.5 kV. The digest was analyzed using the data dependent multitask capability of the instrument acquiring full scan mass spectra to determine peptide molecular weights and product ion spectra to determine amino acid sequence in successive instrument scans. The data were analyzed by using all CID spectra collected in the experiment to search the human reference sequence database using the programs Mascot and Sequest. Protein and peptide identifications were validated with the program Scaffold to a FDR of 0.1%. The proteins identified in the control and IP samples were compared using the total number of spectra identified in the LC-MS/MS analysis. Putative binding partners were identified as those proteins that were only identified in the high and low salt IP samples, by three or more unique peptides, and were absent in the control sample. Data in S1 Table were ranked based on the total spectra count, where the more spectra that were identified, resulted in higher confidence of the identification.

### Functional annotation clustering

Functional annotation clustering of putative CAP-D3 interactors was performed using *DAVID* analysis (https://david.ncifcrf.gov/) [96, 97] with the following parameters: Kappa similarity (term overlap: 3, similarity threshold: 0.50); Classification (initial group membership: 3, final group membership: 3, multiple linkage threshold: 0.50); Enrichment Thresholds (EASE: 1). Benjamini corrected FDR < 0.05 was considered significant.

### RNA analysis by qRT-PCR

Cells were lysed and their total RNA initially extracted with Trizol (Life Technologies), followed by further purification using an RNeasy Mini Kit (Qiagen). cDNA was generated from 1μg of RNA using the TaqMan Reverse Transcription Reagents (Applied Biosystems) and oligo-dT primer. Subsequent RT-qPCR reactions used Fast Start Sybr Green [120] and were run on a Roche Lightcycler 480. Three independent experiments were performed in all cases and results were averaged together. Oligo sequences were as follows:

L1 ORF1 sense: 5’-TCAAAGGAAAGCCCATCAGACTA-3’
L1 ORF1 antisense: 5’-TGGCCCCCACTCTCTTCT-3’
Actin sense: 5’-CCAACCGCGAGAAGATGACC-3’
Actin antisense: 5’-GGAGTCCATCACGATGCCAG-3’

### RNA immunoprecipitation (RIP)-PCR assay for L1 RNA binding

The interaction between CAP-D3, EPRS, eIF4G and L1 RNA in cells was evaluated using a ribonucleoprotein immunoprecipitation assay [94, 121]. Briefly, 6x10^6^ HT-29 cells were seeded onto 150mm dishes and transfected with a wild-type human L1 plasmid, pLRE-*mEGFPI*, using FuGENE 6 (Promega). After 24 h, transfected cells were selected with 10mg/mL of puromycin. On day 2 post-transfection, the cells were washed with PBS and incubated at room temperature with 1% formaldehyde (in PBS) for 10 minutes to crosslink closely juxtaposed RNA-protein constituents. The reaction was quenched by the addition of glycine to 0.125M for an additional 5 min. The cells were then washed twice with cold PBS, recovered from the dishes, pelleted by centrifugation, and stored as pellets at -80°C until use. For RIP, cells were resuspended in 1mL RIPA buffer (300mM NaCl, 50mM Tris pH 7.5, 1mM EDTA, 0.1% Triton, 10% glycerol, 1mM DTT, 1X protease inhibitor cocktail) and dounce homogenized. The lysate was cleared by centrifugation at 14,000 x g for 15 min at 4°C and applied to either uncoated, or antibody coated, Protein A Dynabeads (Thermo Fisher). Briefly, 50μl of beads were incubated with either 4–6μg of antibody for 4 hours at room temperature, and then washed with PBS/BSA solution. Equal volumes of lysate (500μl) were added to antibody coated protein A beads and beads lacking antibody to control for specificity. Beads and lysate were incubated overnight at 4°C, and then washed twice before elution into 80μl of 50mM glycine, pH 2.8 by heating at 70°C for 45 minutes to reverse crosslinks. RNA was then extracted from 50μl of unbound lysate or bound eluate using Trizol-LS reagent (Invitrogen). The RNA was DNase treated using RNase-free DNase (Promega). First strand cDNA was synthesized from 500ng of the recovered RNA using the TaqMan Reverse Transcription Reagents (Applied Biosystems). L1 RNA was detected in the unbound lysates and bound eluates by semi-quantitative PCR using the Advantage 2 PCR kit according to the manufacturer’s recommendation (Clontech Laboratories). Here, PCR products were visualized on a 1.8% agarose gel. L1 RNA was also detected by RT-PCR using Fast Start Sybr Green [120] and run on a Roche Lightcycler 480. Forward and reverse primers specific to the L1-5’UTR, ORF1, EGFP, and the *Neomycin* resistance cassette were utilized. CT values or signal intensity of ethidium bromide stained bands for the bound eluates were normalized to total RNA and the unbound fractions for the same immunoprecipitation and lysate conditions to determine relative binding to the L1 RNA. Oligo sequences were as follows:

L1 5’UTR sense: 5’-ACGGAATCTCGCTGATTGCTA-3’
L1 5’UTR antisense: 5’-AAGCAAGCCTGGGCAATG-3’
L1 ORF1 sense: 5’-TCAAAGGAAAGCCCATCAGACTA-3’
L1 ORF1 antisense: 5’-TGGCCCCCACTCTCTTCT-3’
L1 Neo sense: 5’- CATGCTGCTATAAAGACACATGCAC-3’
L1 Neo antisense: 5’- GTCGTTTGTTCGGATCGGGTTAG-3’
EGFP sense: 5’-CATGGTCCTGCTGGAGTTCGTG-3’
EGFP antisense: 5’-CGTCGCCGTCCAGCTCGACCAG-3’
GAPDH sense: 5’-GCTCTGCATATACTTGATCAGGTCG-3’
GAPDH antisense: 5’-CGAAGATCGGAATTAACGGATTTGGC-3’.

### Polysome profiling

Three million Non-Target or CAP-D3 shRNA expressing cells were seeded into 150mm dishes and treated with IPTG for 48 hours to induce shRNA expression. On the day of the assay, 100μg/mL of cycloheximide was added directly to the culture media and incubated at room temperature for 15 minutes. The cells were then washed twice with PBS and harvested. Ten million cells were lysed in TMK lysis buffer (10mM Tris pH 7.4, 5mM MgCl_2_, 100mM KCl, 1% Triton X-100, 0.5% Deoxycholate, 1U/mL RNase Out, 2mM DTT, and 100μg/mL cycloheximide) and incubated on ice for 20 minutes. The lysate was then cleared by centrifugation at 12,000 RPM for 10 minutes. A 10–50% sucrose gradient was made using an Isco Model Fraction collector and UV Absorbance Monitor in thin-walled centrifuge tubes (Beckman-Coulter) and incubated for 1 hour at 4°C. The lysate was loaded onto the gradient and centrifuged at 17,000 RPM overnight. The data and fractions were collected using the Isco Model Fraction collector and UV Absorbance Monitor. For RNA isolation and purification, Trizol-LS and an RNeasy Mini Kit (Qiagen) were utilized, respectively. Equal volumes of RNA were used in downstream RT-qPCR analyses for ORF1, and Actin levels among the 8 fractions. ORF1 levels were normalized to Actin levels for each fraction and calculated as a percentage of the total RNA in all fractions for that sample, as described in [122].Oligo sequences were as follows:

L1 ORF1 sense: 5’-TCAAAGGAAAGCCCATCAGACTA-3’
L1 ORF1 antisense: 5’-TGGCCCCCACTCTCTTCT-3’
Actin sense: 5’-CCAACCGCGAGAAGATGACC-3’
Actin antisense: 5’-GGAGTCCATCACGATGCCAG-3’

### Statistical analyses

Data are expressed as mean ± SD (*n* = 3 independent experiments performed under identical conditions). Statistical analyses were performed using GraphPad Prism^©^ (GraphPad Software) with an unpaired Student t-test. A *p*-value < 0.05 was considered statistically significant.

## Supporting information

S1 Table Putative CAP-D3 interactors, as determined by CAP-D3 immunoprecipitation in HT-29 cells and subsequent mass spectrometry. Putative interactors are listed in order of binding partners exhibiting the highest total spectral counts. Each protein identified is further described by its gene symbol and GeneInfo Identifier (GI) number. The number of unique peptides is shown for each protein identified under both the low and high salt lysis conditions.
(DOCX)

S1 Fig Retrotransposition assays in cells expressing a second inducible CAP-D3 shRNA. (A) Cells expressing an inducible Non-Target (control) or CAP-D3 shRNA were treated with 1μM IPTG for 48 hours to assess knockdown efficiency of CAP-D3 by immunoblotting. Actin was used as a loading control. (B) Retrotransposition assays involving full-length, retrotransposition competent (wild type) L1 elements in HT-29 cells expressing Non-Target or CAP-D3 CAP-H2 shRNAs (top row). Retrotransposition assays using a retrotransposition-defective L1 ORF1p mutant are represented in the bottom row. Crystal violet stained drug-resistant foci were quantified using ImagePro. (C) Retrotransposition assays involving wild-type L1 (pJM101/L1.3) in mock (PBS) treated HT-29 cells. Crystal violet stained drug-resistant foci were quantified using ImagePro for each condition and quantitation is shown in the chart on the right. P-values were calculated with a student t-test.
(TIF)

S2 Fig Proliferation assays in Non-Target or CAP-D3 shRNA expressing cells. Fluorescence intensity, corresponding to levels of cell proliferation in Non-Target or CAP-D3 shRNA expressing cells measured by the CyQUANT NF assay (n = 2). P-values were calculated with a student t-test.
(TIF)

S3 Fig PCR for RNA IPs with and without reverse transcriptase. RNA-IP assays using no antibody or CAP-D3 antibody in HT-29 cell lysate. Binding of CAP-D3 to the L1 RNA using cDNA prepared with and without reverse transcriptase is shown by ethidium bromide staining.
(TIF)

S4 Fig CAP-D3 co-precipitates EPRS in primary human cells. CAP-D3 immunoprecipitation and immunoblotting for CAP-D3 (top) or EPRS (bottom) in colonic epithelial cells isolated from resected human intestinal tissue. CAP-D3 immunoprecipitations were performed in addition to antibody only (no lysate) and IgG antibody controls.
(TIF)

S5 Fig CAP-D3 co-precipitates phosphorylated EPRS. CAP-D3 immunoprecipitation and immunoblotting for phosphorylated EPRS^Ser886^ in nuclear and cytoplasmic HT-29 cell fractions. CAP-D3 immunoprecipitations were performed in addition to antibody only (no lysate) and IgG antibody controls.
(TIF)

S6 Fig The tRNA synthetase, FARSA, does not regulate L1 expression levels. qRT-PCR and immunoblotting analysis of L1 RNA and ORF1 protein levels in HT-29 cells transfected with FARSA siRNA or control siRNA. Actin was used as a loading control. P-values were calculated with a student t-test. A *p*-value < 0.05 was considered statistically significant.
(TIF)

S7 Fig IFN-γ is not expressed in HT-29 cells. Immunoblotting for IFN-γ using recombinant IFN-γ protein and whole cell lysates from Non-Target or CAP-D3 shRNA expressing HT-29 cells. Actin was used as a loading control.
(TIF)

S8 Fig Depletion of CAP-D3 does not alter protein levels of essential GAIT complex subunits. Immunoblotting analysis of EPRS and L13a protein levels in HT-29 cells transfected with CAP-D3 or control siRNA. Actin was used as a loading control.
(TIF)

S9 Fig Depletion of Condensin II and GAIT subunits results in decreased L1 ORF1p protein levels. (A, B) Immunoblotting analysis of endogenous L1 ORF1 protein in CAP-D3 siRNA transfected HT-29 (A) and RPE-1 cells (B) compared to control siRNA transfected cells. (C) Immunoblotting analysis of endogenous L1 ORF1 protein in EPRS siRNA (left panel) transfected HT-29 cells compared to control siRNA transfected cells. Actin was used as a loading control. The results shown in this figure are repeats of experiments performed in Fig 6.
(TIF)

S10 Fig Controls for eIF4G RNA binding assays. (A-C) Quantitation of RNA-IP assays using no antibody or eIF4G antibody to immunoprecipitate proteins from Non-Target, CAP-D3 (A), CAP-H2 (B), or EPRS (C) shRNA expressing cells. Assays were conducted in cells transfected with an EGFP-tagged L1 construct. Binding of eIF4G to L1 RNA was detected using primers directed against the EGFP sequence and levels were normalized to the signal intensity in the unbound fractions (n = 3). P-values were calculated with a student t-test. A *p*-value < 0.05 was considered statistically significant. (D) Quantitation of RNA-IP assays under no antibody and eIF4G immunoprecipitation conditions from Non-Target or CAP-D3 shRNA expressing cells. Binding of eIF4G to GAPDH RNA using primers directed against GAPDH in the presence and absence of CAP-D3 was normalized to the signal intensity in the unbound fractions (n = 3). P-values were calculated with a student t-test. A *p*-value < 0.05 was considered statistically significant.
(TIF)

S11 Fig Depletion of CAP-D3 does not alter protein levels of the GAIT target, ZIPK. Immunoblotting analysis of ZIPK protein levels in HT-29 cells transfected with CAP-D3 or control siRNA. Actin was used as a loading control.
(TIF)

S12 Fig Predicted GAIT elements exist in both the human L1 sequence and in *Drosophila* retrotransposon sequences. Predicted secondary structures of the full length L1.3 3’UTR (A, left panel), the L1.3 3’UTR present in the pJM101/L1.3 construct used in the experiments presented in this paper (A, right panel), L1 5’UTR (B), and two *Drosophila* retrotransposon 3’UTRs (C), as determined by RNAFold, suggests possible areas that resemble GAIT elements (black arrows) found in inflammatory mRNAs inhibited by the GAIT complex in monocytes (99,119). The minimum free energy structures shown exhibit probable base pairing on a scale from 0–1, with base pairing of 0 shown in blue and 1 shown in red.
(TIF)

S1 Methods Supplementary Methods. (DOCX)
