# Supplementary material for: Condensin II and GAIT complexes cooperate to restrict LINE-1 retrotransposition in epithelial cells
Source: PLoS Genet. 2017 Oct 13;13(10):e1007051. doi: 10.1371/journal.pgen.1007051 (PMC5656329; doi:10.1371/journal.pgen.1007051)
Supplement: S1 Table — Putative interactors are listed in order of binding partners exhibiting the highest total spectral counts. Each protein identified is further described by its gene symbol and GeneInfo Identifier (GI) number. The number of unique peptides is shown for each protein identified under both the low and high salt lysis conditions. (DOCX) [file pgen.1007051.s001.docx]

**Supplementary Table 1. Putative CAP-D3 binding partners identified by LC-MS/MS.**

| **Protein** | **Gene Symbol** | **Accession** | **# Unique Peptides** | |
| --- | --- | --- | --- | --- |
|  |  |  | **Low Salt** | **High Salt** |
| dihydrolipoyllysine-residue succinyltransferase component of 2-oxoglutarate dehydrogenase complex, mitochondrial isoform 1 precursor | DSLT | 19923748 | 187 | 206 |
| myosin-9 | MYH9 | 12667788 | 176 | 287 |
| dihydrolipoyllysine-residue acetyltransferase component of pyruvate dehydrogenase complex, mitochondrial precursor | DLAT | 31711992 | 80 | 70 |
| dihydrolipoyl dehydrogenase, mitochondrial isoform 1 precursor | DLD | 91199540 | 77 | 56 |
| condensin-2 complex subunit D3 | NCAPD3 | 45356151 | 68 | 114 |
| dolichyl-diphosphooligosaccharide--protein glycosyltransferase subunit 1 precursor | RPN1 | 4506675 | 63 | 27 |
| 26S protease regulatory subunit 7 isoform 1 | PSMC2 | 4506209 | 62 | 78 |
| lipoamide acyltransferase component of branched-chain alpha-keto acid dehydrogenase complex, mitochondrial precursor | DBT | 392494079 | 62 | 49 |
| pyruvate dehydrogenase E1 component subunit beta, mitochondrial isoform 1 precursor | PDHB | 156564403 | 58 | 49 |
| elongation factor 1-alpha 1 | EEF1A1 | 4503471 | 55 | 94 |
| pyruvate dehydrogenase E1 component subunit alpha, somatic form, mitochondrial isoform 2 precursor | PDHA1 | 291084742 | 48 | 63 |
| 26S protease regulatory subunit 4 | PSMC1 | 24430151 | 47 | 46 |
| myoferlin isoform a | MYOF | 7305053 | 44 | 18 |
| voltage-dependent anion-selective channel protein 1 | VDAC1 | 4507879 | 43 | 35 |
| 40S ribosomal protein S3 isoform 1 | RPS3 | 15718687 | 40 | 45 |
| ATP synthase subunit beta, mitochondrial precursor | ATP5B | 32189394 | 39 | 38 |
| mitochondrial import receptor subunit TOM70 | TOMM70A | 54607135 | 37 | 8 |
| ATP synthase subunit alpha, mitochondrial isoform a precursor | ATP5A1 | 50345984 | 36 | 28 |
| dnaJ homolog subfamily A member 3, mitochondrial isoform 1 | DNAJA3 | 205360838 | 33 | 32 |
| ATPase family AAA domain-containing protein 3A isoform 2 | ATAD3A | 283436222 | 32 | 13 |
| 40S ribosomal protein S3a isoform 1 | RPS3A | 4506723 | 29 | 30 |
| bifunctional glutamate/proline--tRNA ligase | EPRS | 62241042 | 29 | 23 |
| membrane-associated progesterone receptor component 1 isoform 1 | PGRMC1 | 5729875 | 29 | 19 |
| apoptosis regulator BAX isoform alpha | BAX | 20631958 | 29 | 10 |
| glyceraldehyde-3-phosphate dehydrogenase isoform 1 | GAPDH | 576583519 | 28 | 44 |
| heme oxygenase 2 isoform a | HMOX2 | 555943918 | 28 | 14 |
| ras-related protein Rab-5C isoform b | RAB5C | 354721184 | 27 | 19 |
| 26S protease regulatory subunit 8 isoform 1 | PSMC5 | 24497435 | 26 | 31 |
| voltage-dependent anion-selective channel protein 2 isoform 2 | VDAC2 | 296317339 | 24 | 19 |
| elongation factor 1-gamma | EEF1G | 4503481 | 23 | 47 |
| 26S protease regulatory subunit 6B isoform 1 | PSMC4 | 5729991 | 23 | 35 |
| dolichol-phosphate mannosyltransferase subunit 1 | DPM1 | 4503363 | 23 | 18 |
| ras-related protein Rab-6A isoform a | RAB6A | 19923231 | 23 | 16 |
| vesicle-associated membrane protein-associated protein A isoform 2 | VAMPA | 94721252 | 23 | 14 |
| annexin A2 isoform 2 | ANAX2 | 209862831 | 22 | 44 |
| ADP/ATP translocase 2 | SLC25A5 | 156071459 | 22 | 29 |
| 40S ribosomal protein S5 | RPS5 | 13904870 | 22 | 13 |
| pyruvate dehydrogenase protein X component, mitochondrial isoform 1 precursor | PDHX | 203098753 | 21 | 16 |
| filaggrin-2 | FLG2 | 62122917 | 21 | 9 |
| ras-related protein Rab-10 | RAB10 | 256222019 | 20 | 20 |
| ras-related protein Rab-1A isoform 1 | RAB1A | 4758988 | 20 | 18 |
| vesicle-trafficking protein SEC22b precursor | SEC22B | 380837121 | 20 | 18 |
| 40S ribosomal protein S9 | RPS9 | 14141193 | 20 | 17 |
| 26S proteasome non-ATPase regulatory subunit 10 isoform 1 | PSMD10 | 4506217 | 19 | 23 |
| elongation factor 1-delta isoform 2 | EEF1D | 194239727 | 19 | 18 |
| 60S ribosomal protein L26 isoform X1 | RPL26 | 530410543 | 19 | 3 |
| voltage-dependent anion-selective channel protein 3 isoform 2 | VDAC3 | 208879465 | 18 | 9 |
| dnaJ homolog subfamily C member 13 | DNAJC13 | 112421122 | 18 | 7 |
| sideroflexin-1 | SFXN1 | 23618867 | 17 | 9 |
| alpha-soluble NSF attachment protein | NAPA | 47933379 | 16 | 10 |
| caspase-14 precursor | CASP14 | 6912286 | 16 | 7 |
| acyl-CoA-binding domain-containing protein 5 isoform 2 | ACBD5 | 109689720 | 15 | 19 |
| ATP synthase subunit O, mitochondrial precursor | ATP5O | 4502303 | 15 | 16 |
| ras-related protein Rab-18 isoform 1 | RAB18 | 10880989 | 15 | 14 |
| signal peptidase complex subunit 2 | SPCS2 | 162417971 | 15 | 14 |
| 40S ribosomal protein S8 | RPS8 | 4506743 | 15 | 13 |
| cytochrome c1, heme protein, mitochondrial precursor | CYC1 | 21359867 | 15 | 10 |
| signal recognition particle receptor subunit beta | SRPRB | 284795266 | 15 | 10 |
| U5 small nuclear ribonucleoprotein 200 kDa helicase | SNRNP200 | 40217847 | 15 | 9 |
| 60S ribosomal protein L7 | RPL7 | 15431301 | 15 | 7 |
| 60S ribosomal protein L7a | RPL7A | 4506661 | 15 | 7 |
| 40S ribosomal protein S10 | RPS10 | 323276700 | 15 | 6 |
| NADH-cytochrome b5 reductase 1 | CYB5R1 | 49574502 | 15 | 6 |
| secretory carrier-associated membrane protein 1 isoform 1 | SCAMP1 | 116256358 | 15 | 6 |
| ATP synthase subunit gamma, mitochondrial isoform H (heart) precursor | ATP5C1 | 4885079 | 15 | 5 |
| cytochrome b-c1 complex subunit 1, mitochondrial precursor | UQCRC1 | 46593007 | 14 | 20 |
| ATP synthase F(0) complex subunit B1, mitochondrial precursor | ATP5F1 | 21361565 | 14 | 10 |
| 60S acidic ribosomal protein P2 | RPLP2 | 4506671 | 14 | 9 |
| microsomal glutathione S-transferase 1 isoform a | MGST1 | 9945306 | 14 | 9 |
| atlastin-2 isoform 1 | ATL2 | 208610000 | 14 | 5 |
| myosin regulatory light chain 12B | MYL12B | 15809016 | 13 | 22 |
| suprabasin isoform 1 precursor | SBSN | 260436922 | 13 | 22 |
| 40S ribosomal protein S15a | RPS15 | 14165469 | 13 | 9 |
| NADH-cytochrome b5 reductase 3 isoform 3 | CYB5R3 | 284448551 | 13 | 3 |
| filaggrin | FLG | 60097902 | 12 | 49 |
| 60S ribosomal protein L13 isoform 1 | RPL13 | 15431295 | 12 | 14 |
| transmembrane emp24 domain-containing protein 9 precursor | TMED9 | 39725636 | 12 | 8 |
| BAG family molecular chaperone regulator 2 | BAG2 | 4757834 | 12 | 7 |
| peptidyl-prolyl cis-trans isomerase FKBP11 isoform 3 precursor | FKBP11 | 219842220 | 12 | 6 |
| transmembrane protein 33 isoform X1 | TMEM33 | 530376690 | 12 | 5 |
| 40S ribosomal protein S2 | RPS2 | 15055539 | 12 | 4 |
| heat shock protein beta-1 | HSPB1 | 4504517 | 11 | 40 |
| probable ATP-dependent RNA helicase DDX5 isoform X1 | DDX5 | 530411696 | 11 | 31 |
| ras-related protein Rab-1B | RAB1B | 13569962 | 11 | 12 |
| vesicle-associated membrane protein-associated protein B/C isoform 1 | VAMPB | 4759302 | 11 | 11 |
| 60S ribosomal protein L10 isoform a | RPL10 | 223890243 | 11 | 10 |
| ER membrane protein complex subunit 2 | EMC2 | 7661910 | 11 | 6 |
| protein FAM3C precursor | FAM3C | 7661714 | 11 | 6 |
| ras-related protein Ral-A precursor | RALA | 33946329 | 11 | 6 |
| protein-glutamine gamma-glutamyltransferase E | TGM3 | 189458821 | 10 | 18 |
| histone H3.3 | H3F3B | 4885385 | 10 | 14 |
| adipocyte plasma membrane-associated protein | APMAP | 24308201 | 10 | 11 |
| signal peptidase complex subunit 3 | SPCS3 | 11345462 | 10 | 9 |
| torsin-1A-interacting protein 1 isoform 2 | TOR1AIP1 | 39753957 | 10 | 9 |
| syntaxin-7 | STX7 | 170932494 | 10 | 4 |
| synaptogyrin-2 | SYNGR2 | 4759202 | 9 | 9 |
| probable glutathione peroxidase 8 | GPX8 | 192455698 | 9 | 6 |
| 60S ribosomal protein L14 | RPL14 | 78000183 | 9 | 4 |
| 60S ribosomal protein L24 | RPL24 | 4506619 | 9 | 4 |
| tripartite motif-containing protein 4 isoform beta | TRIM4 | 15011941 | 9 | 4 |
| succinate dehydrogenase [ubiquinone] flavoprotein subunit, mitochondrial | SDHA | 156416003 | 9 | 3 |
| V-type proton ATPase subunit d 1 | ATP6V0D1 | 19913432 | 9 | 3 |
| dolichyl-diphosphooligosaccharide--protein glycosyltransferase 48 subunit precursor | DDOST | 20070197 | 8 | 17 |
| cytochrome c oxidase subunit 4 isoform 1, mitochondrial precursor | COX4I1 | 4502981 | 8 | 13 |
| ras-related C3 botulinum toxin substrate 1 isoform Rac1b | RAC1 | 9845509 | 8 | 8 |
| 60S acidic ribosomal protein P1 isoform 1 | RPLP1 | 4506669 | 8 | 7 |
| serine/arginine-rich splicing factor 1 isoform 2 | SRSF1 | 118582269 | 8 | 4 |
| 40S ribosomal protein S13 | RPS13 | 4506685 | 8 | 3 |
| 60S ribosomal protein L21 | RPL21 | 18104948 | 8 | 3 |
| cytochrome b-c1 complex subunit 2, mitochondrial precursor | UQCRC2 | 50592988 | 7 | 41 |
| tubulin alpha-4A chain isoform 1 | TUBA4A | 17921989 | 7 | 16 |
| serine/threonine-protein phosphatase PGAM5, mitochondrial isoform 1 | PGAM5 | 281604136 | 7 | 15 |
| cytochrome c oxidase subunit 5A, mitochondrial precursor | COX5A | 190885499 | 7 | 8 |
| 60S ribosomal protein L13a isoform 1 | RPL13A | 6912634 | 7 | 6 |
| 60S ribosomal protein L18a | RPL18A | 11415026 | 7 | 6 |
| malectin precursor | MLEC | 7661948 | 7 | 6 |
| NADH dehydrogenase [ubiquinone] 1 beta subcomplex subunit 10 | NDUFB10 | 4758774 | 7 | 6 |
| cytochrome b5 type B | CYB5B | 83921614 | 7 | 5 |
| syntaxin-12 | STX12 | 28933465 | 7 | 5 |
| 60S ribosomal protein L27a | RPL27A | 4506625 | 7 | 4 |
| 60S ribosomal protein L30 | RPL30 | 4506631 | 7 | 4 |
| cytochrome c oxidase subunit 5B, mitochondrial precursor | COX5B | 17017988 | 7 | 4 |
| NADH dehydrogenase [ubiquinone] 1 beta subcomplex subunit 9 isoform 1 | NDUFB9 | 6274550 | 7 | 4 |
| ras-related protein Rab-3D | RAB3D | 4759000 | 7 | 4 |
| OCIA domain-containing protein 1 isoform 1 | OCIAD1 | 119874205 | 7 | 3 |
| protein FAM162A | FAM162A | 49355721 | 7 | 3 |
| ubiquitin carboxyl-terminal hydrolase isozyme L5 isoform X11 | XP_006711433.1 | 578801064 | 7 | 3 |
| elongation factor 2 | EEF2 | 4503483 | 6 | 33 |
| heat shock cognate 71 protein isoform 1 | HSPA8 | 5729877 | 6 | 23 |
| elongation factor 1-beta | EEF1B2 | 11136628 | 6 | 7 |
| synaptophysin-like protein 1 isoform b | SYPL1 | 33239443 | 6 | 7 |
| NADH dehydrogenase [ubiquinone] iron-sulfur protein 8, mitochondrial isoform X1 | NDUFS8 | 530396818 | 6 | 6 |
| cytochrome b-c1 complex subunit Rieske, mitochondrial | UQCRFS1 | 163644321 | 6 | 5 |
| translocon-associated protein subunit alpha precursor | SSR1 | 169404009 | 6 | 5 |
| 2-oxoglutarate dehydrogenase, mitochondrial isoform 3 precursor | OGDH | 259013553 | 6 | 4 |
| 60S ribosomal protein L15 isoform 1 | RPL15 | 15431293 | 6 | 4 |
| tyrosine-protein phosphatase non-receptor type 1 isoform 1 | PTPN1 | 4506289 | 6 | 4 |
| 60S ribosomal protein L18 isoform 2 | RPL18 | 395132436 | 6 | 3 |
| ras-related protein Rab-35 isoform 1 | RAB35 | 5803135 | 6 | 3 |
| structural maintenance of chromosomes protein 4 isoform 1 | SMC4 | 50658063 | 5 | 246 |
| myosin light polypeptide 6 isoform 1 | MYL6 | 17986258 | 5 | 23 |
| desmocollin-3 isoform Dsc3a preproprotein | DSC3 | 148539846 | 5 | 21 |
| DNA-directed RNA polymerase II subunit RPB3 | POLR2C | 14702171 | 5 | 10 |
| T-complex protein 1 subunit gamma isoform a | CCT3 | 63162572 | 5 | 9 |
| RPL17-C18orf32 protein isoform 1 | RPL17-C18orf32 | 313569822 | 5 | 5 |
| uncharacterized protein C2orf47, mitochondrial precursor | C2orf47 | 239582772 | 5 | 5 |
| 60S ribosomal protein L34 isoform X1 | RPL34 | 530378053 | 5 | 4 |
| 60S ribosomal protein L8 | RPL8 | 15431306 | 5 | 4 |
| cytochrome c-type heme lyase | HCCS | 169790849 | 5 | 3 |
| derlin-1 isoform a | DERL1 | 13236516 | 5 | 3 |
| putative deoxyribose-phosphate aldolase | DERA | 116063554 | 4 | 17 |
| tricarboxylate transport protein, mitochondrial isoform a precursor | SLC25A1 | 21389315 | 4 | 7 |
| tumor protein D54 isoform g | TPD52L2 | 345197262 | 4 | 7 |
| nuclease-sensitive element-binding protein 1 | YBX1 | 34098946 | 4 | 6 |
| retinol dehydrogenase 11 isoform 1 precursor | RDH11 | 166795268 | 4 | 6 |
| 40S ribosomal protein S7 | RPS7 | 4506741 | 4 | 5 |
| ER membrane protein complex subunit 8 isoform 1 | EMC8 | 5174615 | 4 | 5 |
| cation-dependent mannose-6-phosphate receptor isoform 1 precursor | M6PR | 4505061 | 4 | 4 |
| ras-related protein Rab-21 | RAB21 | 7661922 | 4 | 3 |
| vesicle-associated membrane protein 3 | VAMP3 | 4759300 | 4 | 3 |
| stomatin-like protein 2, mitochondrial isoform b | STOML2 | 559098406 | 3 | 14 |
| 26S protease regulatory subunit 10B | PSMC6 | 195539395 | 3 | 12 |
| F-actin-capping protein subunit alpha-1 | CAPZA1 | 5453597 | 3 | 10 |
| poly(U)-binding-splicing factor PUF60 isoform b | PUF60 | 17298690 | 3 | 8 |
| nicotinamide phosphoribosyltransferase isoform X1 | NAMPT | 530385393 | 3 | 7 |
| HLA class I histocompatibility antigen, Cw-1 alpha chain precursor | HLA-C | 339882741 | 3 | 5 |
| RNA binding motif protein, X-linked-like-1 | RBMXL1 | 21361809 | 3 | 5 |
| proteasomal ubiquitin receptor ADRM1 isoform X1 | ADRM1 | 530417788 | 3 | 4 |
| succinate dehydrogenase [ubiquinone] iron-sulfur subunit, mitochondrial precursor | SDHB | 115387094 | 3 | 3 |
| condensin-2 complex subunit G2 | NCAPG2 | 116812586 | 2 | 5 |
