# Supplementary figures and images for: Condensin II and GAIT complexes cooperate to restrict LINE-1 retrotransposition in epithelial cells
Source: PLoS Genet. 2017 Oct 13;13(10):e1007051. doi: 10.1371/journal.pgen.1007051 (PMC5656329; doi:10.1371/journal.pgen.1007051)

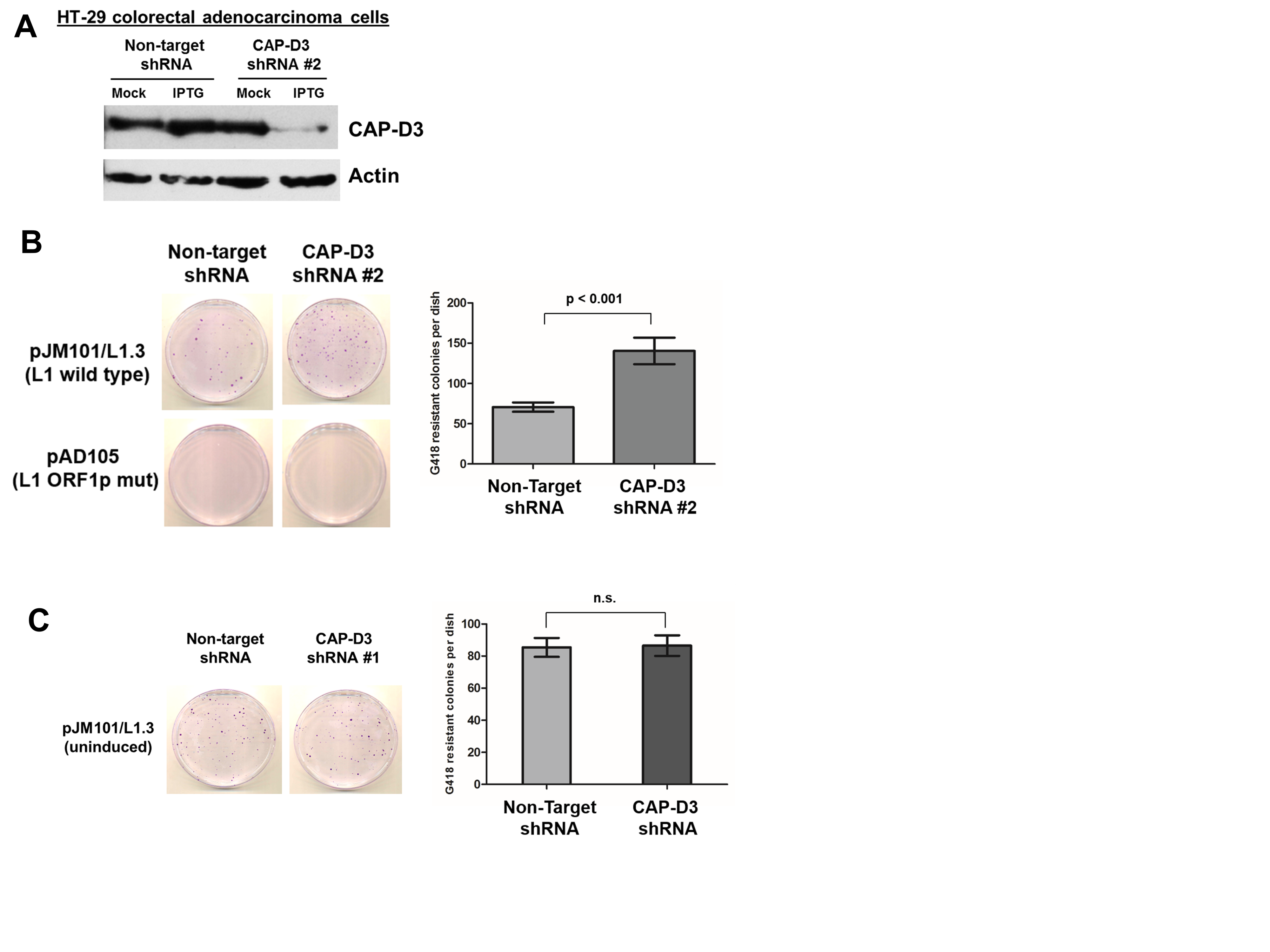

Supplement: S1 Fig — (A) Cells expressing an inducible Non-Target (control) or CAP-D3 shRNA were treated with 1μM IPTG for 48 hours to assess knockdown efficiency of CAP-D3 by immunoblotting. Actin was used as a loading control. (B) Retrotransposition assays involving full-length, retrotransposition competent (wild type) L1 elements in HT-29 cells expressing Non-Target or CAP-D3 CAP-H2 shRNAs (top row). Retrotransposition assays using a retrotransposition-defective L1 ORF1p mutant are represented in the bottom row. Crystal violet stained drug-resistant foci were quantified using ImagePro. (C) Retrotransposition assays involving wild-type L1 (pJM101/L1.3) in mock (PBS) treated HT-29 cells. Crystal violet stained drug-resistant foci were quantified using ImagePro for each condition and quantitation is shown in the chart on the right. P-values were calculated with a student t-test. (TIF) [file pgen.1007051.s002.TIF]

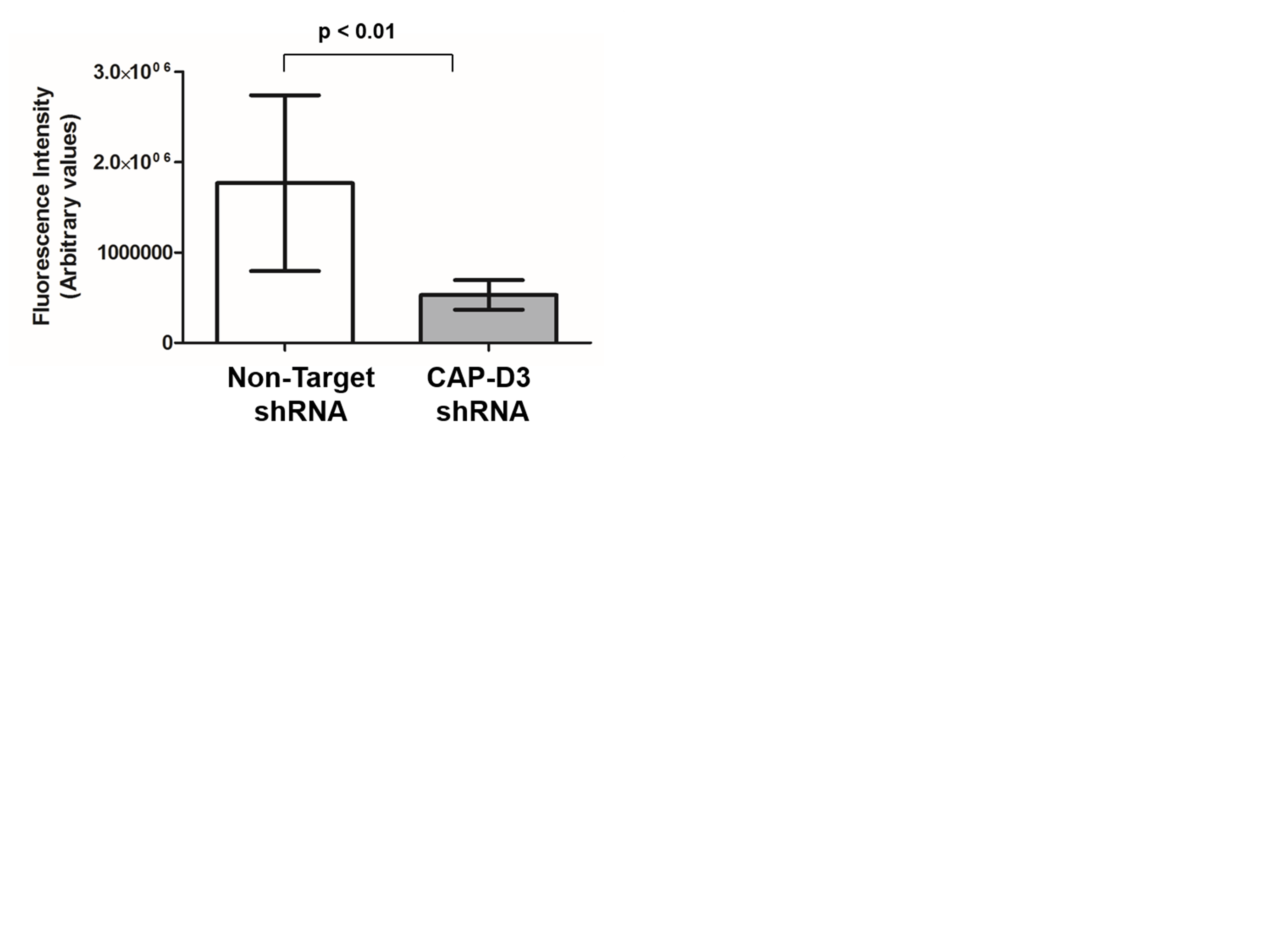

Supplement: S2 Fig — Fluorescence intensity, corresponding to levels of cell proliferation in Non-Target or CAP-D3 shRNA expressing cells measured by the CyQUANT NF assay (n = 2). P-values were calculated with a student t-test. (TIF) [file pgen.1007051.s003.TIF]

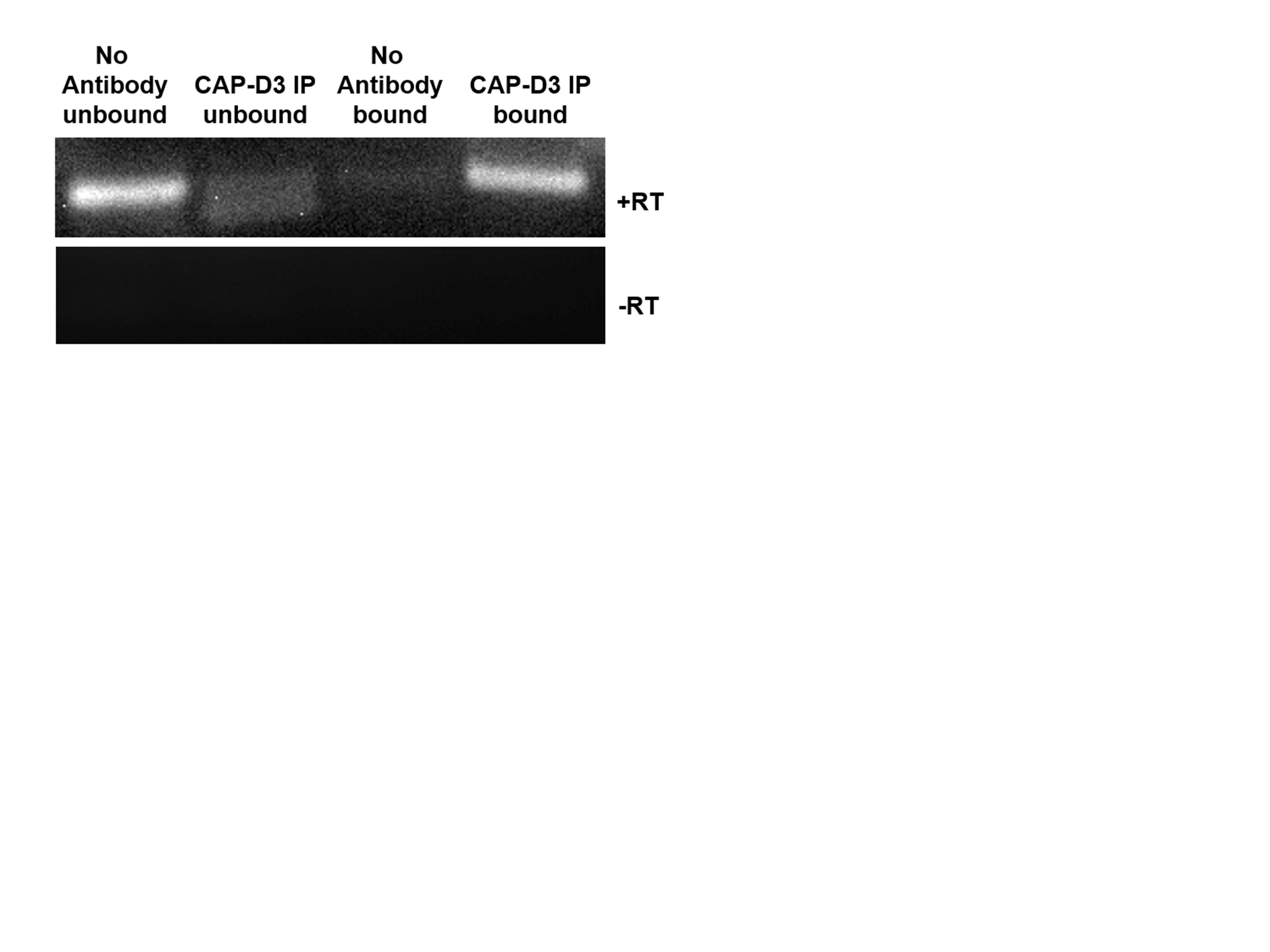

Supplement: S3 Fig — RNA-IP assays using no antibody or CAP-D3 antibody in HT-29 cell lysate. Binding of CAP-D3 to the L1 RNA using cDNA prepared with and without reverse transcriptase is shown by ethidium bromide staining. (TIF) [file pgen.1007051.s004.TIF]

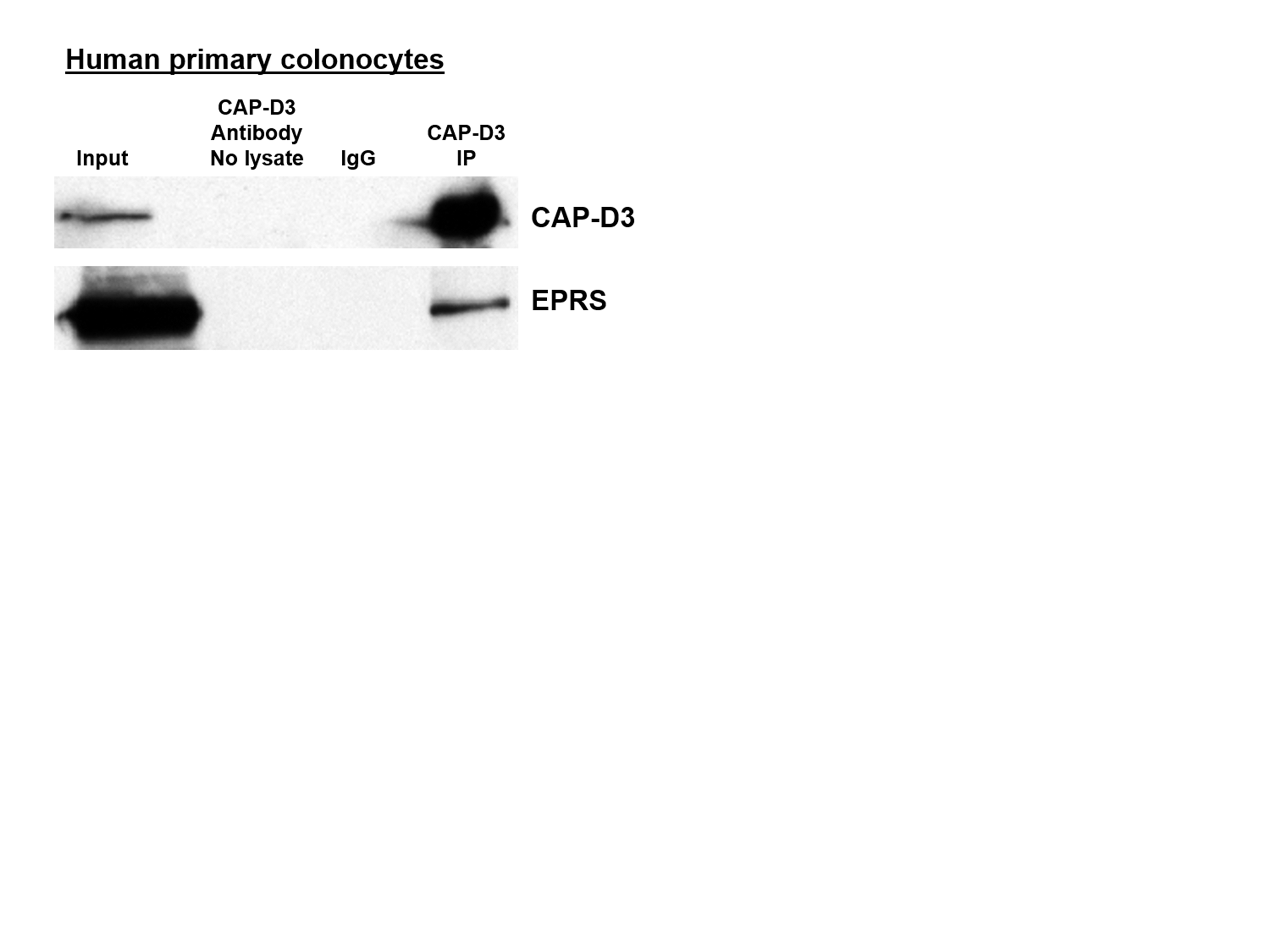

Supplement: S4 Fig — CAP-D3 immunoprecipitation and immunoblotting for CAP-D3 (top) or EPRS (bottom) in colonic epithelial cells isolated from resected human intestinal tissue. CAP-D3 immunoprecipitations were performed in addition to antibody only (no lysate) and IgG antibody controls. (TIF) [file pgen.1007051.s005.TIF]

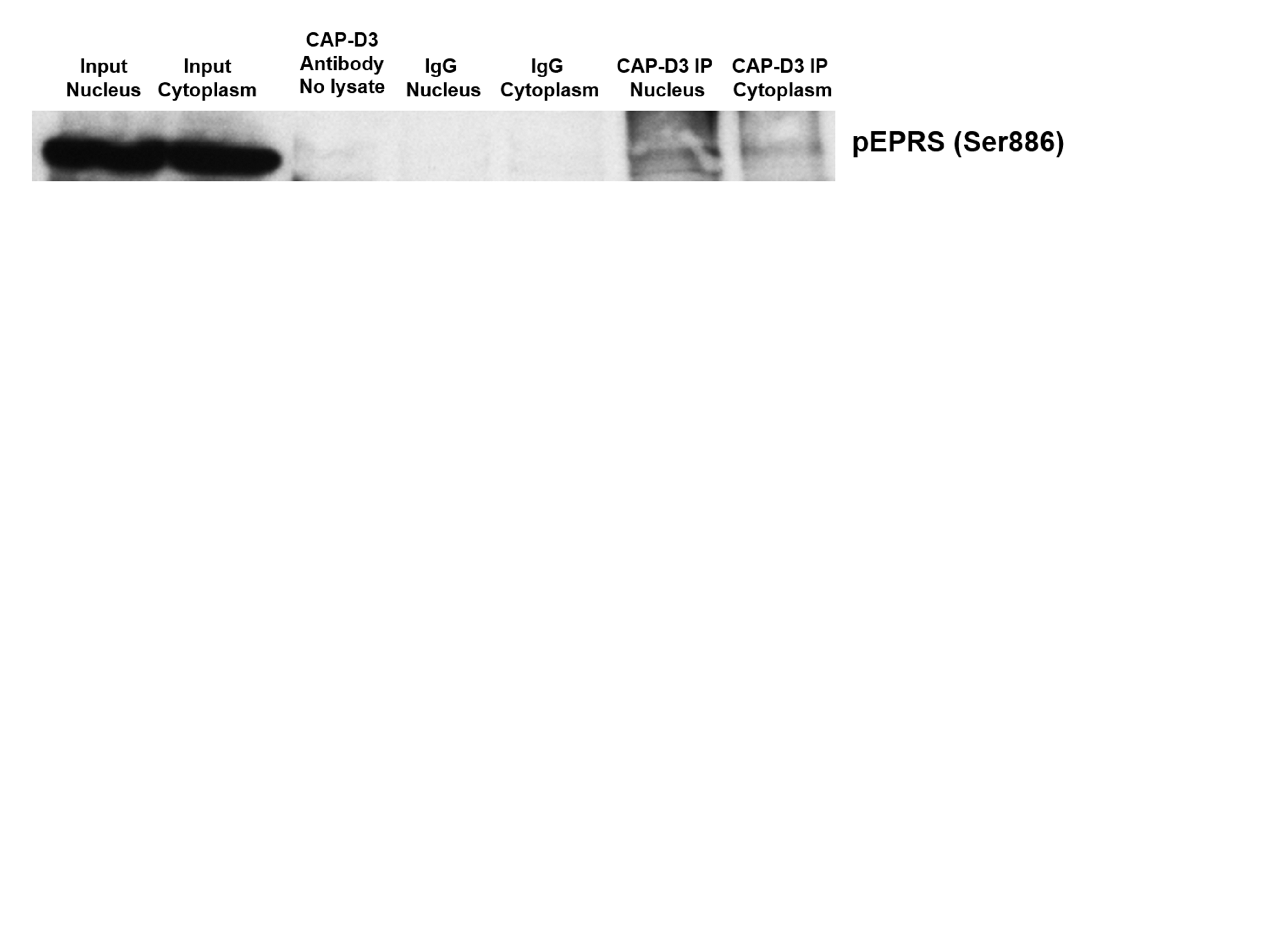

Supplement: S5 Fig — CAP-D3 immunoprecipitation and immunoblotting for phosphorylated EPRSSer886 in nuclear and cytoplasmic HT-29 cell fractions. CAP-D3 immunoprecipitations were performed in addition to antibody only (no lysate) and IgG antibody controls. (TIF) [file pgen.1007051.s006.TIF]

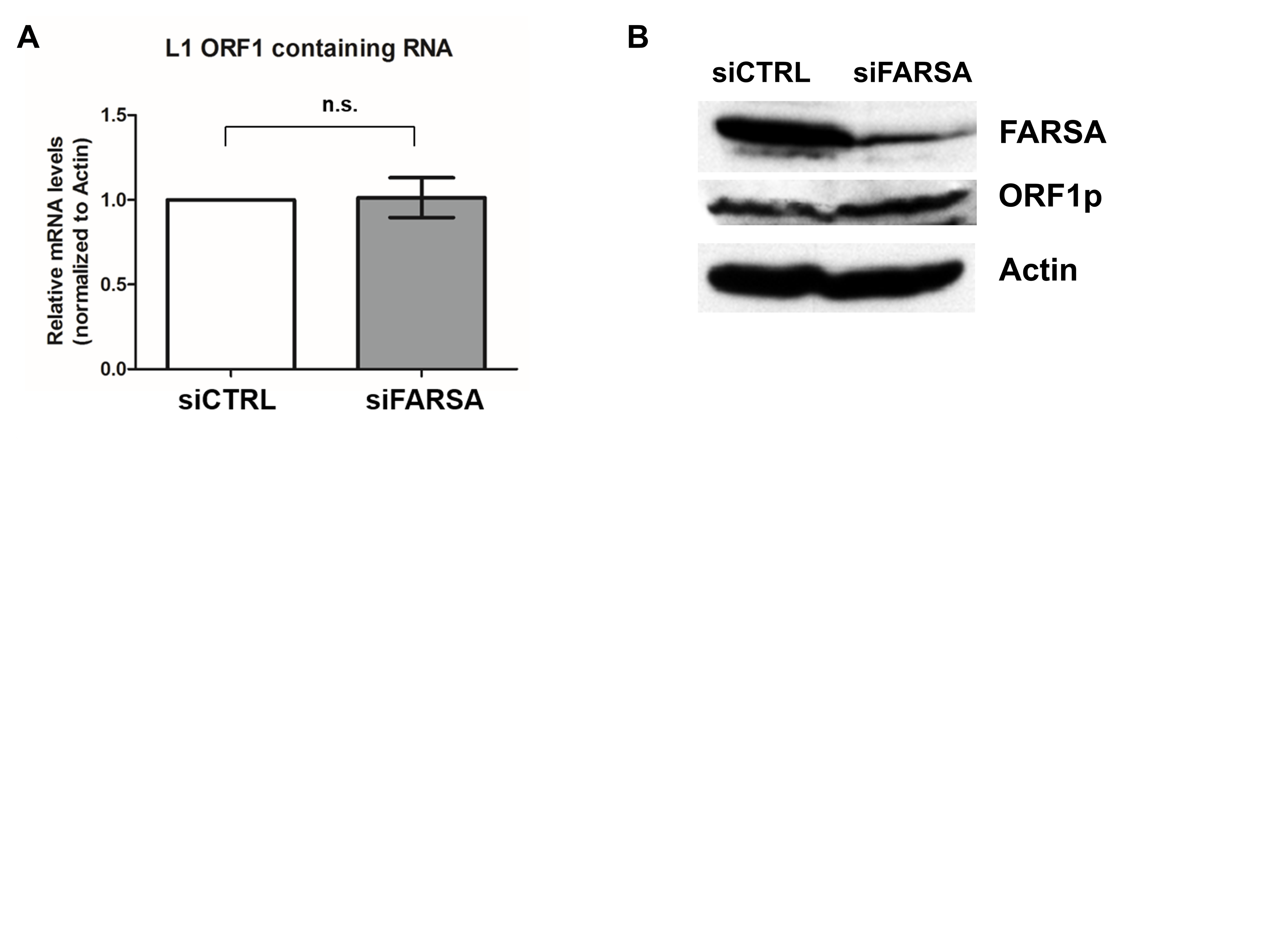

Supplement: S6 Fig — qRT-PCR and immunoblotting analysis of L1 RNA and ORF1 protein levels in HT-29 cells transfected with FARSA siRNA or control siRNA. Actin was used as a loading control. P-values were calculated with a student t-test. A p-value < 0.05 was considered statistically significant. (TIF) [file pgen.1007051.s007.TIF]

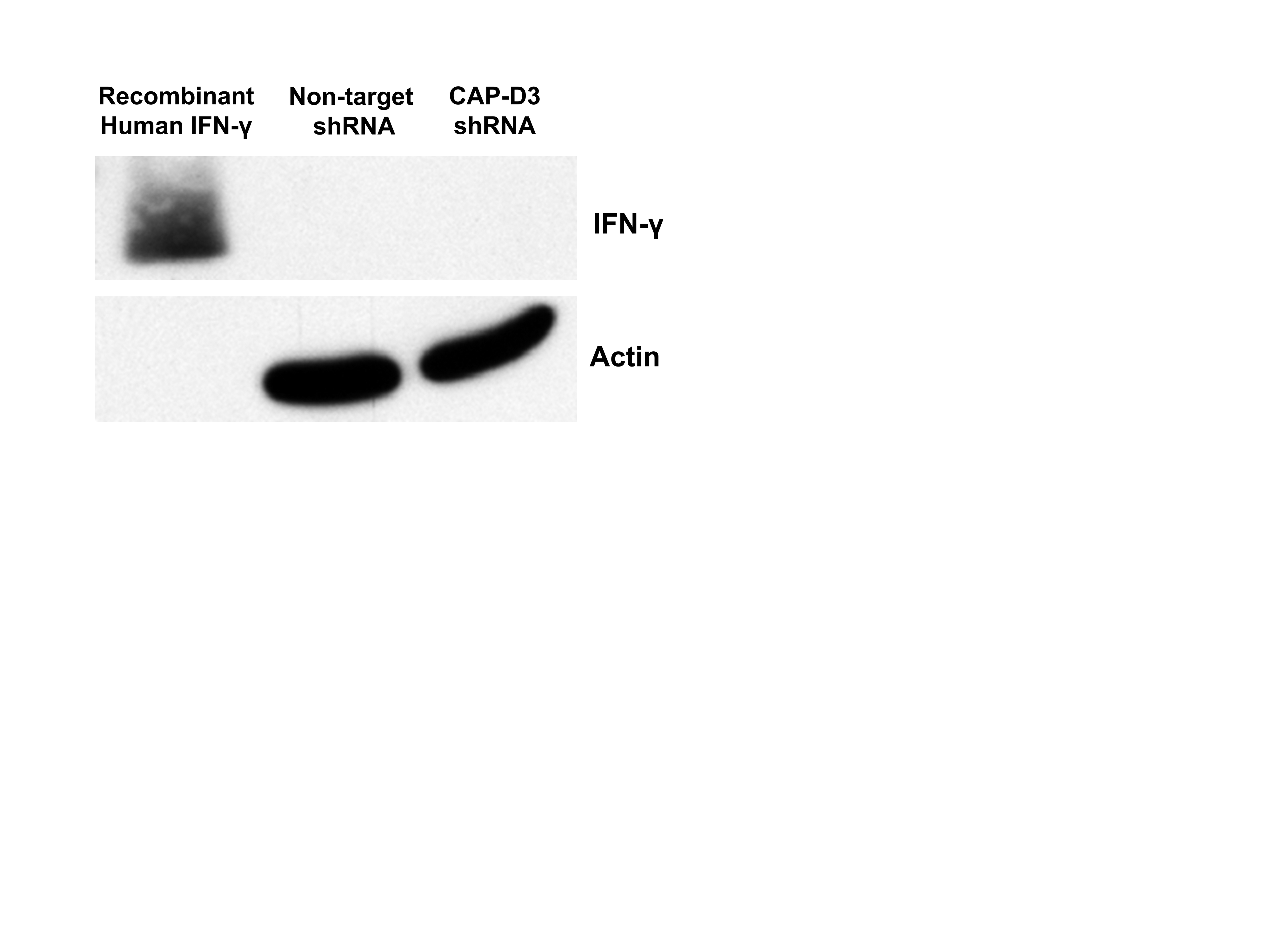

Supplement: S7 Fig — Immunoblotting for IFN-γ using recombinant IFN-γ protein and whole cell lysates from Non-Target or CAP-D3 shRNA expressing HT-29 cells. Actin was used as a loading control. (TIF) [file pgen.1007051.s008.TIF]

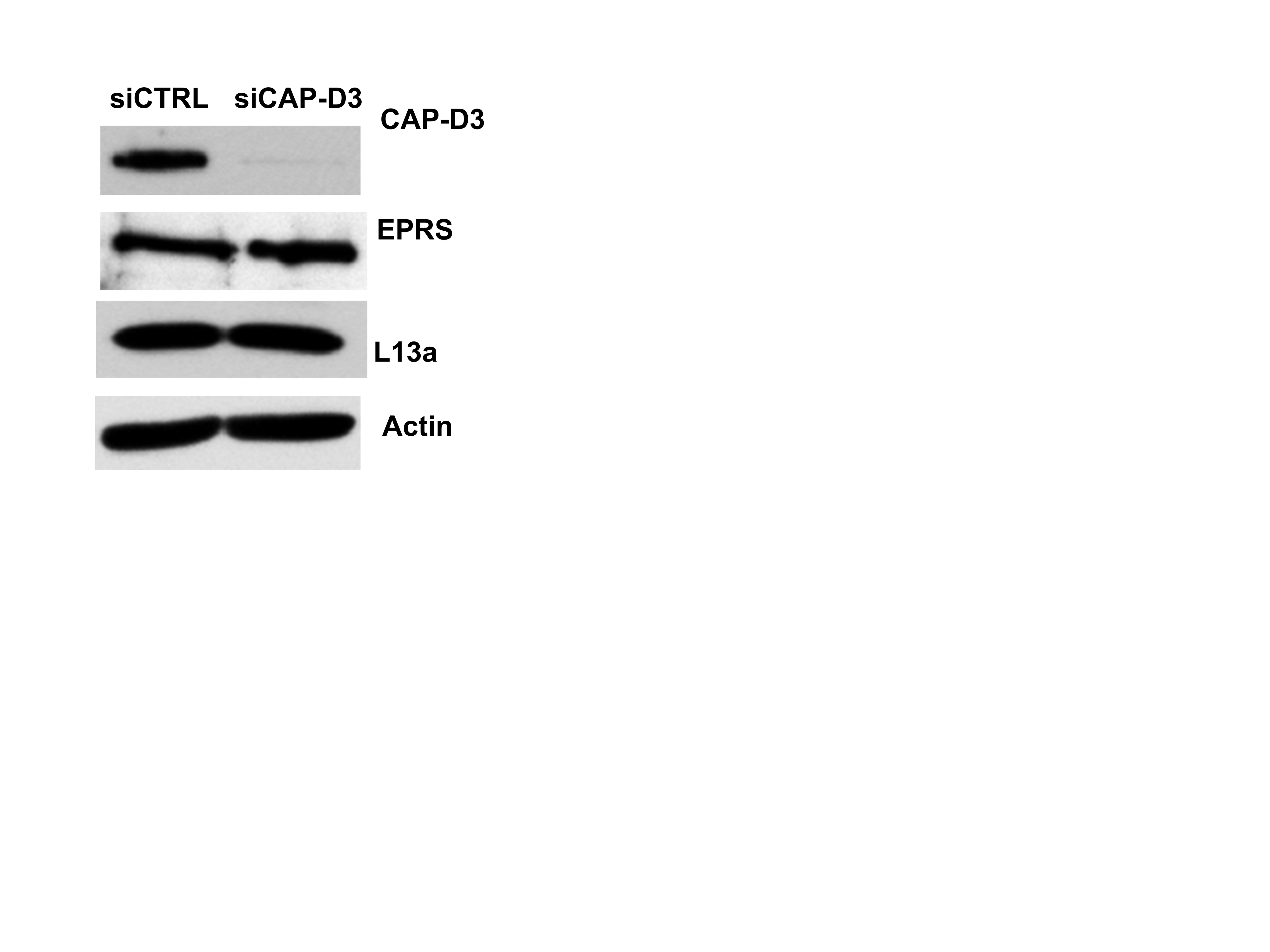

Supplement: S8 Fig — Immunoblotting analysis of EPRS and L13a protein levels in HT-29 cells transfected with CAP-D3 or control siRNA. Actin was used as a loading control. (TIF) [file pgen.1007051.s009.TIF]

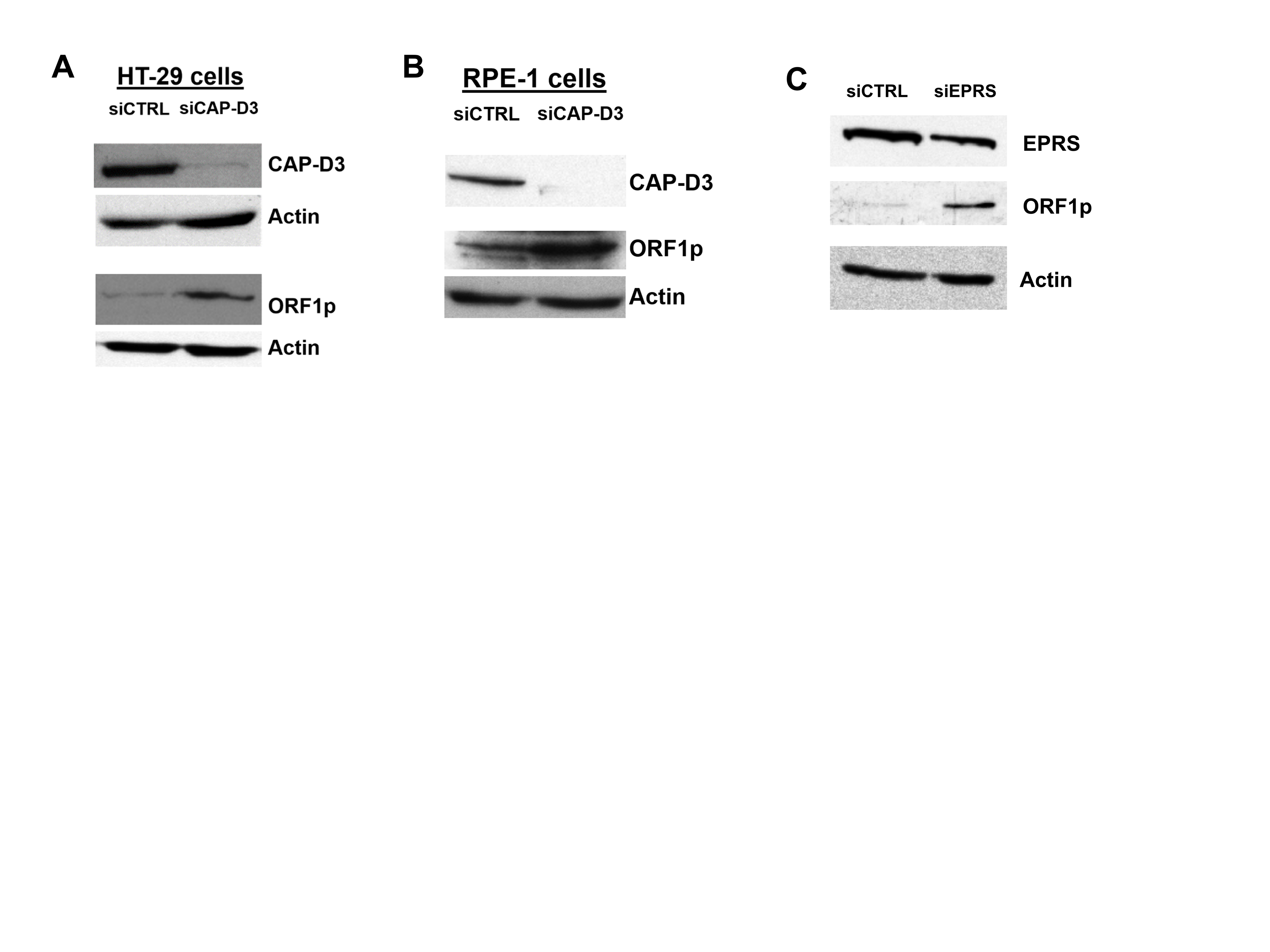

Supplement: S9 Fig — (A, B) Immunoblotting analysis of endogenous L1 ORF1 protein in CAP-D3 siRNA transfected HT-29 (A) and RPE-1 cells (B) compared to control siRNA transfected cells. (C) Immunoblotting analysis of endogenous L1 ORF1 protein in EPRS siRNA (left panel) transfected HT-29 cells compared to control siRNA transfected cells. Actin was used as a loading control. The results shown in this figure are repeats of experiments performed in Fig 6. (TIF) [file pgen.1007051.s010.TIF]

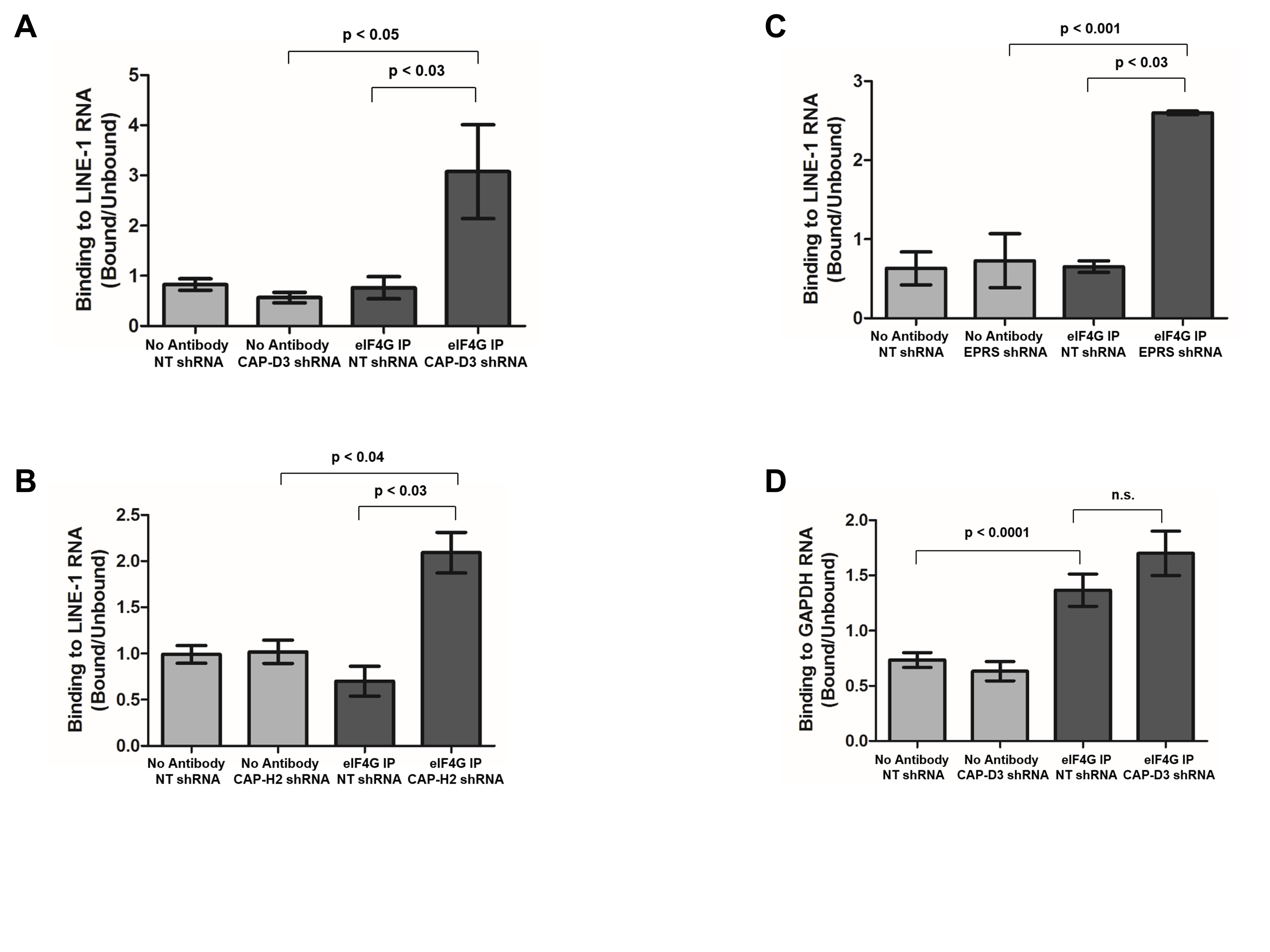

Supplement: S10 Fig — (A-C) Quantitation of RNA-IP assays using no antibody or eIF4G antibody to immunoprecipitate proteins from Non-Target, CAP-D3 (A), CAP-H2 (B), or EPRS (C) shRNA expressing cells. Assays were conducted in cells transfected with an EGFP-tagged L1 construct. Binding of eIF4G to L1 RNA was detected using primers directed against the EGFP sequence and levels were normalized to the signal intensity in the unbound fractions (n = 3). P-values were calculated with a student t-test. A p-value < 0.05 was considered statistically significant. (D) Quantitation of RNA-IP assays under no antibody and eIF4G immunoprecipitation conditions from Non-Target or CAP-D3 shRNA expressing cells. Binding of eIF4G to GAPDH RNA using primers directed against GAPDH in the presence and absence of CAP-D3 was normalized to the signal intensity in the unbound fractions (n = 3). P-values were calculated with a student t-test. A p-value < 0.05 was considered statistically significant. (TIF) [file pgen.1007051.s011.TIF]

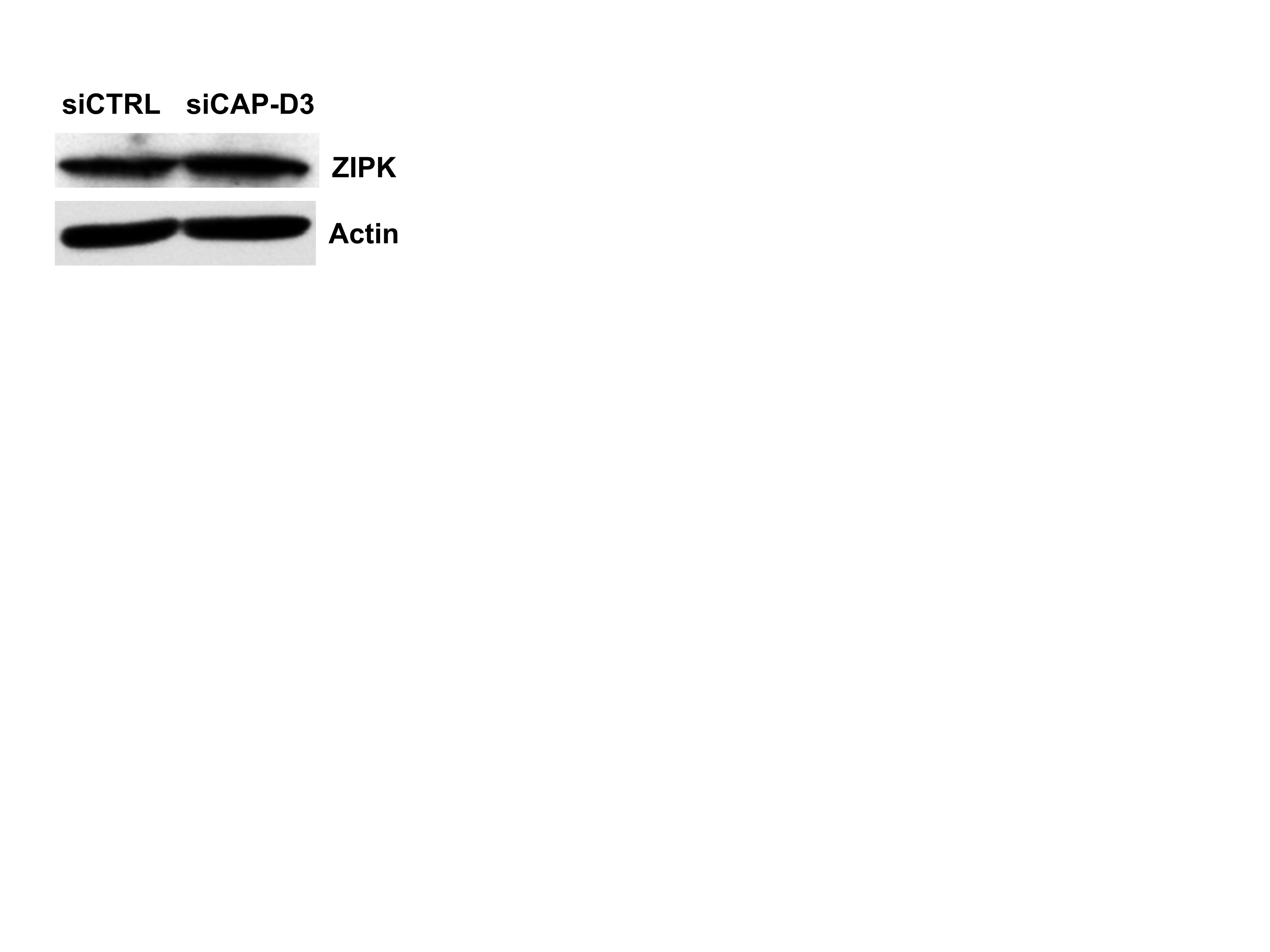

Supplement: S11 Fig — Immunoblotting analysis of ZIPK protein levels in HT-29 cells transfected with CAP-D3 or control siRNA. Actin was used as a loading control. (TIF) [file pgen.1007051.s012.TIF]

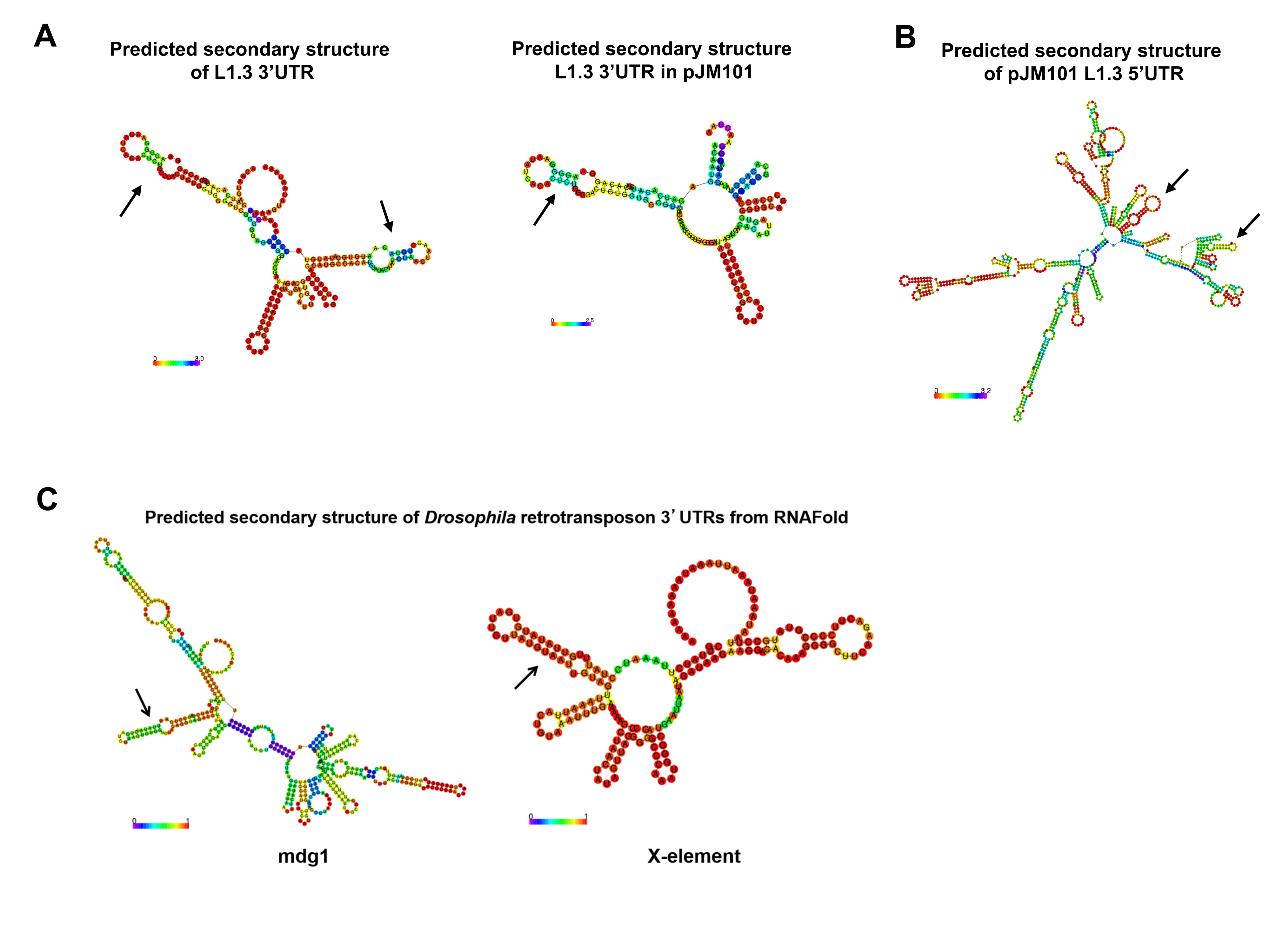

Supplement: S12 Fig — Predicted secondary structures of the full length L1.3 3’UTR (A, left panel), the L1.3 3’UTR present in the pJM101/L1.3 construct used in the experiments presented in this paper (A, right panel), L1 5’UTR (B), and two Drosophila retrotransposon 3’UTRs (C), as determined by RNAFold, suggests possible areas that resemble GAIT elements (black arrows) found in inflammatory mRNAs inhibited by the GAIT complex in monocytes (99,119). The minimum free energy structures shown exhibit probable base pairing on a scale from 0–1, with base pairing of 0 shown in blue and 1 shown in red. (TIF) [file pgen.1007051.s013.TIF]
