# Supplementary material for: Condensin II and GAIT complexes cooperate to restrict LINE-1 retrotransposition in epithelial cells
Source: PLoS Genet. 2017 Oct 13;13(10):e1007051. doi: 10.1371/journal.pgen.1007051 (PMC5656329; doi:10.1371/journal.pgen.1007051)
Supplement: S1 Methods — (DOCX) [file pgen.1007051.s014.docx]

**CyQUANT NF proliferation assay**

Non-Target and CAP-D3 shRNA expressing cells (~5 x10^3^) were seeded into a 96-well plate and IPTG treated for 48 hours. The assay was then performed according to the manufacturer’s protocol. Briefly, the growth media was removed and 100μL of 1X dye binding solution was dispensed into each well and the cells were incubated at 37°C for 60 minutes. This incubation period is required for equilibration of dye–DNA binding, resulting in a stable fluorescence endpoint. Following the incubation the fluorescence intensity of each sample was measured using a Victor^2^ fluorescence microplate reader (PerkinElmer, Waltham, MA) with excitation at ~485 nm and emission detection at ~530 nm. Background fluorescence was corrected for using well without cells present.
